# Photon Recycling in Semiconductor Thin Films and Devices

**DOI:** 10.1002/advs.202004076

**Published:** 2021-08-19

**Authors:** Zhongkai Cheng, Deirdre M. O'Carroll

**Affiliations:** ^1^ Department of Chemistry and Chemical Biology Rutgers University 123 Bevier Road Piscataway NJ 08854 USA; ^2^ Department of Materials Science and Engineering Rutgers University 607 Taylor Road Piscataway NJ 08854 USA

**Keywords:** optoelectronic devices, photon recycling, semiconductor materials

## Abstract

Photon recycling (PR) plays an important role in the study of semiconductor materials and impacts the properties of their optoelectronic applications. However, PR has not been investigated comprehensively and it has not been demonstrated experimentally in many different kinds of semiconductor materials and devices. In this review paper, first, the authors introduce the background of PR and describe how this phenomenon was originally identified in semiconductors. Then, the theory and modelling of PR is reviewed and some of the important parameters that are used to quantify PR are highlighted. Next, a variety of the methods used to achieve and characterize PR in materials and devices are discussed. Examples of how the performance parameters of different types of optoelectronic devices are affected by PR are described. Finally, a summary of the roles of PR in semiconductor materials and devices and an outlook on how PR can be used to solve existing problems and challenges in the field of optoelectronics are provided. From this review, it is apparent that PR can have a positive impact on optoelectronic device performance, and that further in‐depth theoretical and experimental studies are needed to rigorously demonstrate the advantages and importance of PR.

## Graphical abstract

Photon recycling (PR), whereby photons emitted by radiative recombination are reused in semicondcutor materials, significantly influences the optical and electrical properties of semiconductors and the performance of optoelectronic devices. Here, the theory, methods for characterization and implementation of PR in a variety of semiconductors and optoelectronic devices are reviewed.

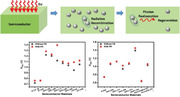

## 1 Introduction

**Figure 1:**
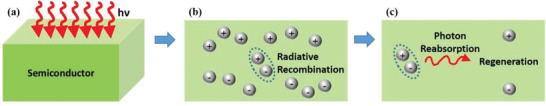
Schematic of the process of PR showing: a) incident photon absorption; b) generation of electrons and holes, and radiative recombination; c) reabsorption of an emitted photon from a radiatively recombined electron‐hole pair with subsequent regeneration of electrons and holes.

It is known that the conversion of light into electricity in a quantum system starts with absorption of a photon via the formation of an excited electronic state. This excited state is used to produce electron‐hole pairs in semiconductor materials and devices.^[^ ^1^, ^2^ ^]^ Electron–hole pairs can separate into mobile electrons and holes that move to different sides of a semiconductor device under an in‐built or external potential. However, some of the electrons and holes recombine either radiatively or nonradiatively. In the case of radiative recombination, the excitation energy is given up in the form of emitted luminescent photons within a device. While the emitted photons often exit the device and radiate into free space, in some instances, the emitted luminescent photons can be reabsorbed by the semiconductor materials and regenerate electron–hole pairs; see **Figure** **1**. This process is called self‐absorption, photon reabsorption or photon recycling (PR).

Radiative recombination is mainly associated with band‐to‐band recombination in semiconductors as a result of the high energy differences associated with a complete band gap transition. It is worth noting that the mechanism by which charge carriers recombine is a factor that determines the lifetime of the charge carrier; just as for direct band gap absorption, direct radiative recombination is a faster process than indirect radiative recombination, which involves more interactions.^[^ ^1^, ^3^, ^4^ ^]^ The factor of how long charge carriers survive before recombination, known as the carrier lifetime, is important and comes into play a role when discussing the ability for charge carriers to contribute to current flow.^[^ ^5^, ^6^, ^7^ ^]^

In 1994, Reuter and Schmitt reported that, in solar cells, radiative recombination depends on the band gap structure of the semiconductor material, on the applied voltage, and on the temperature of both the solar cell and the radiation.^[^ ^8^, ^9^ ^]^ However, as a result of PR, a part of the radiation produced by radiative recombination is not lost and does not escape from the device because the photons are reflected back into the device and are reabsorbed by the semiconductor to produce electron‐hole pairs again.^[^ ^8^, ^10^ ^]^ Therefore, once a luminescent photon is emitted internally as a result of radiative recombination, it will have one of several possible destinies: 1) it can emit into free space through the light escape cone formed by the interface of the device and the external environment (e.g., air); 2) it can be reabsorbed by parasitic optical losses in the device structure; 3) it can be absorbed in the active semiconductor material and experience reincarnation as an electron–hole pair or as a photon again.^[^ ^11^ ^]^ Therefore, efficient PR in semiconductor devices requires significant reabsorption of radiatively emitted photons in the semiconductor material, which implies low parasitic absorption losses and high light confinement within the device at energies corresponding to the band gap.^[^ ^12^ ^]^ This may be achieved using a variety of strategies to confine photon emission in semiconductors, including, for example, incorporation of the semiconductor in an optical cavity or positioning the semiconductor near plasmonic nanomaterials. Additionally, increasing the extent to which photon emission couples to guided modes of semiconductor thin films can also sufficiently trap the radiatively emitted photons so that the photons can be absorbed one or more times by the semiconductor.

Until now, PR has been observed and achieved in a range of semiconductor devices whose active materials include: crystalline silicon materials;^[^ ^13^, ^14^, ^15^, ^16^ ^]^ direct band gap III‐V semiconducting materials;^[^ ^17^, ^18^, ^19^, ^20^, ^21^, ^22^, ^23^, ^24^, ^25^, ^26^ ^]^ quantum dots materials;^[^ ^27^ ^]^ and perovskite materials.^[^ ^28^, ^29^, ^30^ ^]^ This phenomenon has received considerable attention because of its role in modifying carrier lifetime and its prevalence in semiconductor materials that exhibits high quantum yields and large optical absorption coefficients.^[^ ^5^, ^31^ ^]^ There are two classic methodologies to describe and evaluate the influence of PR on the performance of different semiconductor devices. One method approaches the problem by introducing a carrier lifetime enhancement factor to describe the average shift in carrier parameters as a result of photon reabsorption.^[^ ^26^, ^31^ ^]^ Another is based on the modification of the continuity equations for the electric charge and complicates the problem of solving the standard semiconductor equations because PR appears as a non‐linear term that depends on the geometry of the device and its operating conditions.^[^ ^31^, ^32^, ^33^ ^]^ In this review paper, we discuss the theory of the PR effect and methods to achieve and characterize PR. We also review numerous examples of PR in different semiconductor materials and in different types of optoelectronic devices.

## 2 Theory of Photon Recycling

Since the concept of PR was first introduced in the 1950s to the present day, the theory of PR has become more complete due to a large amount of long‐term research for a variety of semiconductor materials and devices. Initially, when PR was found to be only related to radiative recombination, carrier diffusion and carrier lifetime were regarded as only two factors affecting PR. At that time, PR was easy to model and the materials available for research were limited, such as Si and Ge. However, as the definition of PR becomes clear, more and more influencing factors were discovered and expressed, such as PR factor and PR generation rate. On the one hand, the advantages are that the theory of PR is not as confusing and convoluted as before and it can be studied from different entry points, which makes the research more flexible and enhances innovation. On the other hand, many factors may also be more complicated, which would increase the difficulty of the theoretical study. Nowadays, in addition to the traditional inorganic semiconductor materials and devices, some new materials like perovskites have also been used in the theoretical study of PR. Therefore, although the theory of PR still needs a long time to develop and study, the influence of PR in different materials and devices has already received extensive attention because it is very important that it is beneficial to control PR in materials and devices to exhibit good performance.

PR consists of the reabsorption of photons generated in a semiconductor device as a result of radiative recombination. As a consequence of this process, also known as photon self‐absorption or self‐excitation, new electron‐hole pairs are generated and the carrier population becomes altered. Actually, the phenomenon of self‐excitation or PR based on radiative recombination was first identified and studied in 1957.^[^ ^5^, ^34^, ^35^ ^]^ At that time, this effect received considerable attention in the field of semiconductors because of its role in the enhancement of carrier diffusion and carrier lifetime. However, the basic foundations of the theory of PR start with the principles of radiative recombination. In 1954, Van Roosbroeck and Shockley evaluated quantities characterizing the radiative recombination process, such as the thermal equilibrium recombination rate at room temperature, the recombination cross section and the decay time for a small disturbance in carrier concentration in the intrinsic Ge material.^[^ ^6^ ^]^ Their study was based on the principle of detailed balance, whereby the rate of radiative recombination at thermal equilibrium is equal to the corresponding rate of generation of electron‐hole pairs by thermal radiation.^[^ ^6^ ^]^ They represented the total rate of radiative recombination, *R*, at thermal equilibrium as follows:

(1) $$R=\int P\left(v\right)\rho \left(v\right)\mathrm{dv}$$

where

(2) $$P\left(v\right)\rho \left(v\right)=\frac{\left(\frac{32\pi^{2}\kappa n^{3}}{c^{3}}\right)\;v^{3}}{\exp\left(\frac{\mathrm{hv}}{\mathrm{kT}}\right)-1}$$

and *P*(*v*) is the probability of absorption of a photon of frequency, *v*, per unit time, *ρ*(*v*) is the thermal equilibrium distribution of photon density in the material, *κ* is absorption index, *n* is refractive index, *c* is the speed of light, *h* is Planck constant, *k* is the Boltzmann constant and *T* is temperature. From this study it was concluded that for Ge, radiative and nonradiative recombination are responsible for the long and short carrier lifetimes, respectively.^[^ ^5^, ^6^ ^]^ Although Equation (2) has limitations in that it was applied to a specific material (Ge) under room temperature, it provided a foundation for quantifying radiative recombination in later studies.

Subsequently, in 1957, Dumke derived expressions for radiative recombination lifetimes based on a microscopic analysis of both direct and indirect band gap transitions in crystalline semiconductors.^[^ ^5^ ^]^ Dumke applied these expressions to crystalline Ge and compared their results with the radiative lifetimes calculated by the method of Van Roosbroeck and Shockley to test the consistency of the calculations and of the physical model on which they are based. Dumke's work made an advancement over the prior work of Van Roosbroeck and Shockley because it is shown that in crystalline Ge at room temperature, the rate of recombination by direct transitions is greater than that by indirect transitions. Dumke concluded that for semiconductors which have a high absorption constant, an emitted photon is usually reabsorbed to produce another electron–hole pair rather than contributing to the macroscopically observed radiative lifetime.^[^ ^5^ ^]^ This scenario alters the diffusion of electron‐hole pairs and, hence, the carrier lifetime. Therefore, this research was the first to indicate the existence of the PR effect and that it was promoted by strong absorption close to the band gap in direct band gap semiconductors.^[^ ^33^, ^36^ ^]^ According to the findings from Dumke that it is necessary to account for PR effects in direct band gap semiconductors, it is supposed that the observed radiative recombination lifetime would be infinite if all the photons emitted by the direct recombination of the hole–electron pairs are reabsorbed within the semiconductor. However, it is not achievable because some of the emitted radiation is generated close to the surfaces of the materials and escapes before the reabsorption process occurs. Thus, the radiative lifetime has a finite value that is dependent on the thickness of the semiconductor layer and the optical properties of the surfaces. These initial studies depended on fundamental knowledge and provided effective methods and approaches for the future theoretical research in PR.

### 2.1 Photon Recycling Factor

**Figure 2:**
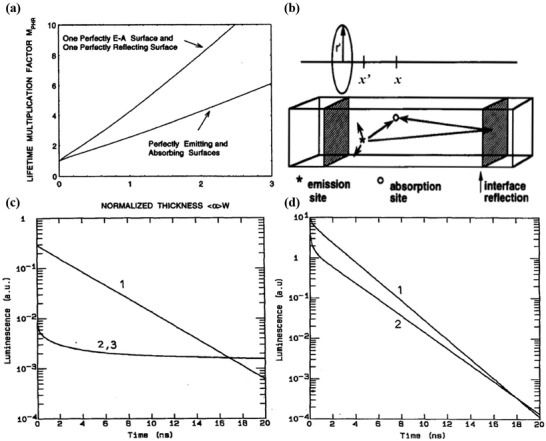
a) PR factor, *M*_PHR_ (also known as the radiative lifetime multiplication factor), as a function of normalized active layer thickness for two different scenarios. b) Visualization of the PR process in an example double heterostructure and cylindrical coordinate system employed. c) Calculated luminescence decay after a light excitation pulse in GaAs‐Ga_x_Al_1‐__x_As double heterostructure. Curve 1: neglecting PR; Curve 2: calculated with the general term of PR through Equation (7); Curve 3: calculate with added correction on *τ* and *D* through Equation (6). d) Calculated luminescence decay of a typical GaAs diode. Curve 1: without PR; Curve 2: with PR. a) Reproduced with permission.^[^ ^36^ ^]^ Copyright 2001, AIP Publishing. b) Reproduced with permission.^[^ ^38^ ^]^ Copyright 1994, IEEE. c,d) Reproduced with permission.^[^ ^37^ ^]^ Copyright 1992, AIP Publishing.

In 1977, Asbeck was the first author to determine a PR factor through quantitative analysis of the effects of self‐absorption of spontaneously emitted photons on the recombination lifetime of injected carriers in the active layer of GaAs‐GaAlAs double heterostructure.^[^ ^26^, ^36^ ^]^ In subsequent work, Borrego et al. introduced the PR factor (*M*_PHR_), also known as the lifetime multiplication factor, into an expression for effective total lifetime (*τ*_eff_) of a semiconductor measured in a photon conductive decay measurement that takes into account the PR effect as follows:^[^ ^36^ ^]^

(3) $$\frac{1}{\tau_{\mathrm{eff}}}=\;\frac{1}{\tau_{\mathrm{SRH}}}+\;\frac{1}{M_{\mathrm{PHR}}\tau_{R}}+\;\frac{1}{\tau_{A}}+\;\frac{2S}{W}$$

where *τ*_SRH_ is the Shockley–Read–Hall (SRH) lifetime, *τ*_R_ is the radiative recombination lifetime, *τ*_A_ is the Auger recombination lifetime, *W* is the thickness of the semiconductor layer, and *S* is the surface recombination velocity. Then, based on the research of Kuriyama et al. and Rehaud et al.,^[^ ^32^, ^37^ ^]^ Borrego et al. expressed the general form of the PR factor as:^[^ ^36^ ^]^

(4) $$M_{\mathrm{PHR}}=\frac{2\langle \alpha \rangle WN}{1-2E_{3}\left(N\langle \alpha \rangle W\right)}$$

where *<α>* is the effective absorption coefficient (the average value of the absorption constant *α*), *E*_3_ is the exponential integral function of order 3 and *N* is the number of internal reflections. When there is low PR, the value of *<α>W* (the normalized thickness) is very small and *M*_PHR_ approaches 1. When there is significant PR, the value of *<α>W* becomes large and, in that case, *M*_PHR_ approaches 2*<α>WN*. If both surfaces of the epitaxial layer are considered to be blackbody surfaces (perfectly emitting/absorbing), *N* = 1. However, if one of the surfaces is perfectly reflecting and the other is perfectly absorbing, then *N* = 2, the effective normalized thickness doubles, and the value of *M*_PHR_ becomes twice as large (meaning the radiative lifetime is increased by a factor of two). **Figure** **2**a shows a graph representing the effect of both scenarios on *M*_PHR_. In general, *M*_PHR_ is inversely related to the escaping fraction of generated photons. If all photons escape the active layer, then *M*_PHR_ = 1 and if none escape, then *M*_PHR_ is infinite. Therefore, *M*_PHR_ plays an important role in representing the increase of effective radiative lifetime through the PR processes. Durbin et al. provided another expression for *M*_PHR_, which they referred to as the radiative lifetime multiplication factor (*φ*), that accounts for PR effects in solar cells. The radiative lifetime multiplication factor *φ* is defined as:^[^ ^38^ ^]^

(5) $$\varphi \;=\;\Delta n/\left(\Delta n-G_{\mathrm{PR}}\tau_{R}\right)$$

where Δ*n* is the excess electron density and *G*_PR_ is the PR generation rate (a perturbation term). Therefore, the PR factor/lifetime multiplication factor is a usefulquantification to assess the degree to which PR impacts radiative and total lifetimes and it can be related to the additional electron–hole pair generation rate caused by PR.

### 2.2 Photon Recycling Generate Rate

In 1979, Roedel and Keramidas defined PR as photoluminescence (PL) resulting from the self‐absorption of photons generated by prior luminescence processes. Note this early definition is slightly different to the modern‐day definition in that it assumes the reabsorbed photons create new PL. In this study, they showed that PR affected the steady‐state characteristics, such as the steady‐state electroluminescence spectra, of graded‐band‐gap Ga_1‐__x_Al_x_As:Si light‐emitting diodes (LEDs) strongly and constant‐band‐gap GaAs:Si LEDs weakly.^[^ ^39^ ^]^ In their report, a simplified one‐dimensional (1D) model for PR using the diffusion equation was developed. They wrote the continuity equation in the form:

(6) $$D\frac{d^{2}\Delta n}{dx^{2}}\;\;-\frac{\Delta n}{\tau }\;+g\left(x\right)=0$$

where *D* is the diffusion coefficient, *τ* is the total electron lifetime, and *g(x)* is a term that describes the rate of minority‐carrier generation (per unit distance) due to photon absorption and includes both direct photon absorption and indirect photon reabsorption (i.e., PR). In general, Equation (6) is relevant to all optoelectronic devices.

In 1991, a further development technique demonstrated by Renaud et al. was used to provide a simple evaluation of the effective total recombination lifetime (i.e., radiative and nonradiative) and diffusion coefficient.^[^ ^37^ ^]^ They expressed the continuity equation of minority carriers with the inclusion of PR as:^[^ ^32^, ^37^, ^39^, ^40^ ^]^

(7) $$D\frac{d^{2}\Delta n}{dx^{2}}-\frac{\Delta n}{\tau }-\frac{d\Delta n}{\mathrm{dt}}+\alpha_{e}\Phi \left(t\right)e^{-\alpha_{e}x}+G_{\mathrm{PR}}=0$$

From Equation (7), it is obvious that this equation represents the excitation of a sample along the *x* axis. In this equation, *Φ*(*t*) is the external generation rate where *t* is time, and *α*_e_ is an average absorption coefficient of external photons that takes into account the spectral density of incoming light. Meanwhile, the PR generation rate, *G*_PR_, is expressed in the case of partial confinement of photons as:^[^ ^32^, ^37^, ^39^, ^41^ ^]^

(8) $$G_{\mathrm{PR}}=\frac{\alpha_{i}}{2\tau_{r}}\int_{0}^{W}K\;\left(x,x^{\prime }\right)\Delta n\;\left(x^{\prime },t\right)dx^{\prime }$$

This equation represents the excitation of a sample with thickness *W*, along the *x* axis, where *α*_i_ is the average value of the absorption coefficient over the internal luminescence spectrum, *Δn*(*x,t*) is the number of injected carriers per unit of distance and time, *K*(*x,x’*) is a term that accounts for light trapping and multiple internal reflections and *τ*_r_ is the radiative lifetime. Clearly, based on Equation (8), PR generation rate is larger when absorption and sample thickness are large, and when radiative lifetime is short. For example, Figure 2c,d illustrate that the luminescence of both GaAs‐Ga_x_Al_1‐__x_As double heterostructure and typical GaAs diode with PR decayed faster than those without PR.

In 2006, Balenzategui and Martí presented a model to simulate PR effects which is specifically dedicated to calculating its influence on the optoelectronic device performance, especially on a solar cell's performance.^[^ ^31^ ^]^ The formal modelling, which uses the drift‐diffusion model governed by Poisson to study carrier dynamics in solar cells, is extended to allow explicitly for the occurrence of PR as follows:

(9) $$\frac{\mathrm{dn}}{\mathrm{dt}}=+\frac{1}{q}\nabla \cdot J_{n}+{\left(G_{n}-R_{n}\right)}_{\mathrm{NR}}+\left(G_{\mathrm{Th}}-R_{R}\right)+G_{E}+G_{\mathrm{PR}}$$

(10) $$\frac{\mathrm{dp}}{\mathrm{dt}}=-\frac{1}{q}\nabla \cdot J_{p}+{\left(G_{p}-R_{p}\right)}_{\mathrm{NR}}+\left(G_{\mathrm{Th}}-R_{R}\right)+G_{E}+G_{\mathrm{PR}}$$

where *n* and *p* are the electron and hole concentrations, *q* is elementary charge, ▽ is the gradient, **J**_n_ and **J**_p_ are the drift‐diffusion currents of electrons and holes, (*G* − *R*)_NR_ stands for the net generation rate resulting from nonradiative processes, *G*_E_ is the generation rate merely produced by an external illumination source, *G*_Th_ represents the thermal generation rate associated to photons in equilibrium within the semiconductor and *R*_R_ here is the radiative recombination rate, which is symbolized by *R* in Equation (1). Equations (9) and (10) are the time‐dependent continuity equations for minority carrier densities.^[^ ^31^, ^42^, ^43^, ^44^ ^]^ The PR generation rate is significant in the cases when radiative recombination is the dominant process in the materials and devices.^[^ ^31^ ^]^ The main contribution of the work by Balenzategui and Martí was in computing the extra generation rate produced by PR (i.e., *G*_PR_) by means of a differential equation describing the variation of photon flux density along a given trajectory within the device. The methodology for the calculation based on the use of commercially available programs for semiconductor device simulation provided by Balenzategui and Martí is important because it allows finer details derived from the operating conditions of every point of the device to be introduced into the simulation. It was found that the dark current density of the device reduces when radiative recombination processes are dominant in the device operation and when the PR model has been applied to the simulation of the dark current density‐voltage (*J*–*V)* curve of a GaAs solar cell with a passive substrate.^[^ ^45^, ^46^ ^]^ Thus, the research by Balenzategui and Martí was an advance over previous methods because their model was broader in scope and could be employed for calculating the influence of PR on other semiconductor devices and structures.^[^ ^31^, ^45^, ^46^ ^]^ Although theoretical studies of PR are still in their infancy and the results need to be further confirmed experimentally, the models indicate that PR can exist not only in crystalline semiconductor materials but also in a variety of different semiconductor optoelectronic devices.

### 2.3 Photon Recycling Efficiency

Another metric for evaluating the impact of PR on device performance is PR efficiency, which is the ratio of the number of reabsorbed photons to the total number of emitted photons. Guo et al. calculated the total efficiency of a PR dichromatic light source through a LED device, which consisted of a GaInN/GaN LED emitting in the blue spectral range and an AlGaInP PR semiconductor emitting at the complementary color and has two emission lines, one in the blue (448 nm) and another in the amber (569 nm) wavelength range.^[^ ^47^ ^]^ In this device, a fraction of the blue light emitted from the GaInN/GaN LED was absorbed by the AlGaInP active layer and re‐emitted as lower energy photons. They expressed the total PR efficiency (*η*_PR_) of the PR dichromatic light source as:

(11) $$\eta_{\mathrm{PR}}=\frac{\left(1+R^{\prime }\right)}{\left(\frac{1}{r_{1}}+\frac{R^{\prime }}{r_{1}r_{2}}\frac{\lambda_{2}}{\lambda_{1}}\right)}$$

where *r*_1_ is the electrical‐to‐optical power conversion efficiency (PCE) of the primary LED and *r*_2_ is the optical‐to‐optical PCE of the PR light source.^[^ ^47^ ^]^ Firstly, they defined the power ratio, *R*′, of the two light sources as:

(12) $$R^{\prime }=\frac{P_{2}}{P_{1}}$$

where *P*_1_ and *P*_2_ are the optical powers of the short wavelength source (*λ*_1_) and the long wavelength source (*λ*_2_), respectively. Furthermore, using this formula, they calculated the luminous performance of LED devices as a function of the primary wavelength. Thus, in this case, they determined that the maximum efficiency occurs when the primary wavelength source emits at 440 nm. However, it is noted that both light sources were assumed to emit monochromatic light.

In 2000, Numai analyzed the PR effect through the application of semiconductor lasers. In that report, it was explained that the PR efficiency in laser cavities can be examined by ray tracing.^[^ ^48^ ^]^ It is known that the efficiency of PR is high if the lifetime of spontaneously emitted photons is long. Thus, for the number of rays, *N*′, he defined the PR efficiency, *η*_PR_as:

(13) $$\eta_{\mathrm{PR}}=\frac{1}{N^{\prime }}\sum_{i}^{N^{\prime }}\left[1-\exp\left(-\alpha_{i}^{\prime }L_{i}\right)\right]$$

where *i* corresponds to each optical ray which has its own direction, $\alpha_{i}^{\prime }$ is the power absorption coefficient and *L_i_* is the maximum length at which optical rays satisfy the total internal reflection condition. In addition, subscript *i* in Equation (13) indicates each photon. It is shown that PR improves current versus light output characteristics in semiconductor lasers and does not degrade the modulation speed. According to the Equation (13), PR efficiency significantly depends on the values of *L_i_* and $\alpha_{i}^{\prime }$. In the study by Numai, an absorption coefficient of $\alpha_{i}^{\prime }$ = 20 cm^−1^ was used for all spontaneously emitted rays. Thus, high PR efficiency resulted from the long distance of photon travel in a laser cavity. Compared with a square ring cavity and a triangular ring cavity, it was considered that a circular ring cavity has the largest PR efficiency. However, a square ring cavity, which has a higher PR efficiency than a triangular ring cavity, was determined to be the best performing cavity overall because the number of oscillation modes was less than that of the circular one.^[^ ^48^ ^]^

**Figure 3:**
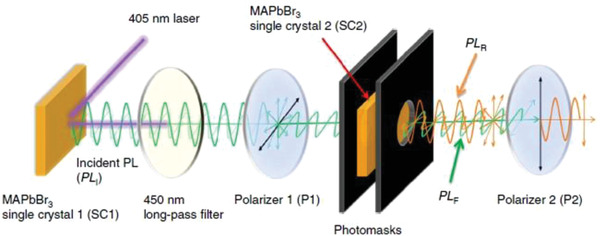
The schematic diagram of the measurement setup of PR efficiency in perovskite single crystals. *PL*_F_ is the intensity of filtered PL. Reproduced with permission.^[^ ^28^ ^]^ Copyright 2017, Nature Publishing Group.

More recently, Fang et al. determined PR efficiency in hybrid perovskite single crystals by measuring the ratio of the re‐emitted photons to the total incident exciting photons.^[^ ^28^ ^]^ The PR efficiency, *η*_PR_, calculated in this case was based on the Equation (14) below:

(14) $$\eta_{\mathrm{PR}}=\frac{2PL_{R}}{\frac{\Omega }{4\pi }PL_{I}\left(1-R^{\prime \prime }\right)}$$

where *PL*_R_ is the intensity of recycled PL (that PL generated by multiple cycles of self‐absorption and re‐emission), *PL*_I_ is the intensity of incident PL, Ω is the critical solid angle into which the photons of *PL*_R_ emitted and *R*′′ is the reflectance of the single crystal surface. PL (*PL*_R_ and *PL*_I_) emissions were measured with the setup schematically shown in **Figure** **3**. It is apparent that PR efficiency is increased when both the recycled emission intensity and the reflectance of the surface is high. However, in their case, the PR efficiencies are revealed to be <0.5% in CH_3_NH_3_PbI_3_ and CH_3_NH_3_PbBr_3_ single crystals under excitation intensity close to one sun, highlighting the intrinsically long carrier recombination lifetime instead of the photon‐recycling‐induced photon propagation as the origin of their long carrier diffusion length. This research is important because it indicates that PR is not limited to traditional inorganic crystalline materials and to theory only. It is possible that PR can be studied theoretically in a variety of materials and their applications, including organic polymers, quantum dots, and organic‐inorganic hybrid materials. In addition to the aforementioned studies, theoretical research of PR in a range of semiconductor materials and devices is needed for a more accurate and complete understanding of PR, particularly in polycrystalline and amorphous semiconductor materials where theoretical studies are currently lacking.

## 3 Methods to Achieve Photon Recycling

According to the concept and theory of the PR effect, there is no doubt that this effect is able to enhance absorption and improve the performance of semiconductor materials and devices. The development of methods to achieve PR has attracted considerable research interests to maximize the performance of different type of devices with this effect. Although there are many methods to achieve PR, in general, four methods are commonly reported: 1) use of thick semiconductor active layers; 2) addition of a rear‐reflector mirror; 3) addition of nanostructures to the active layer; and 4) use of nanostructured back mirrors. In this section, we review some of these common methods for promoting PR in semiconductors. We summarize the methods to achieve PR through different methods in different applications in **Table** **1**. In addition, **Table** S1, Supporting Information clarifies the advantages and disadvantages of each method. We consider the use of nanostructured back mirrors as the most effective method to achieve PR because it can not only enhance light harvesting and trapping in the materials or devices, but it can also increase the propagation distance of emitted photons for a second or multiple pass at absorption. However, the fabrication process is more complicated than other methods. In contrast, although using thick active layers for PR is easier to achieve, this approach conflicts with the next generation of optoelectronics fabricated using thin‐film semiconductors such as organic conjugated polymers or perovskites, because high‐efficiency devices require the active layers to be a few hundred nanometers or less in thickness. Furthermore, nanostructured back mirrors are promising because a variety of different nanostructure sizes and geometries that can be studied, such as nanoholes and nanogratings, in addition to the nanoparticles that have already been reported. These nanostructures can be controlled by physical or chemical methods, such as changing the temperature of dewetting or using different chemical etching materials. Therefore, we believe that nanostructures will play increasingly more important roles in controlling PR in thin‐film semiconductor materials and devices in the future.

**Table 1:** Summary of methods to achieve PR in a variety of semiconductor materials and devices

Method	Active materials	Device type	Reference no.
Thick film	Crystalline Si	Solar cell	^[^ ^15^ ^]^
	AlGaAs/GaAs	LED	^[^ ^50^ ^]^
Rear‐reflector mirror	AlGaAs/GaAs	Reflection modulator	^[^ ^51^ ^]^
	Alq3	LED	^[^ ^54^ ^]^
	AlGaAs/GaAs	LED	^[^ ^55^ ^]^
	AlGaInP	LED	^[^ ^56^ ^]^
	InGaN/GaN	LED	^[^ ^57^ ^]^
	AlGaInP	LED	^[^ ^58^ ^]^
	GaAs	Solar cell	^[^ ^59^ ^]^
	GaAs	Solar cell	^[^ ^60^ ^]^
	GaAs	Solar cell	^[^ ^61^ ^]^
	GaAs	Solar cell	^[^ ^62^ ^]^
	GaAs	Solar cell	^[^ ^63^ ^]^
	GaAs	Solar cell	^[^ ^64^ ^]^
	GaAs	Solar cell	^[^ ^65^ ^]^
	GaAs	Solar cell	^[^ ^66^ ^]^
	GaAs	Solar cell	^[^ ^71^ ^]^
	GaAs	Solar cell	^[^ ^72^ ^]^
Photonic nanostructure	Dye‐sensitized materials	Solar cell	^[^ ^81^ ^]^
	Perovskite	Solar cell	^[^ ^87^ ^]^
Nanostructured rear‐reflector mirror	GaAs GaAs Perovskite	Solar cell Solar cell Solar cell	^[^ ^67^ ^]^ ^[^ ^94^ ^]^ ^[^ ^95^ ^]^

**Note**: Alq3 = Tris(8‐hydroxyquinolinato)aluminum.

### 3.1 Thick Films

The PR effect can be achieved in semiconductor devices through increasing the thickness of the semiconductor active layer because the photons produced by radiative recombination are more likely to undergo reabsorption by the active layer. First‐generation crystalline silicon solar cells exhibited PR effects due to the thick active layers, which have long optical absorption distances. For example, Kerr et al. proposed a new parameterization for Auger recombination in crystalline silicon at 300 K.^[^ ^15^ ^]^ In this work, the effects of active layer thickness, dopant density and dopant type on the limiting efficiency were investigated, including the effects of PR, resulting in a maximum achievable PCE of 29.05% for a 90 µm‐thick silicon active layer fabricated on high resistivity silicon.^[^ ^15^ ^]^ Ettenberg and Kressel determined that the interfacial radiative recombination velocities of Al_0.25_Ga_0.75_As/GaAs and Al_0.5_Ga_0.5_As/GaAs heterojunctions are 4 × 10^3^ and 8 × 10^3^ cm s^−1^, respectively, for heterojunction thickness > 1 µm. On the other hand, for double‐heterojunction thickness < 1 µm, the minority‐carrier lifetime remains constant, suggesting that the interfacial radiative recombination velocity decreases with decreasing heterojunction thickness.^[^ ^49^ ^]^ Furthermore, Asbeck illustrated that the net radiative lifetime of carriers in standard GaAs/Ga_1‐__x_Al_x_As heterostructures with a thick active layer (> 1 µm) may be lengthened by PR effects, which was in good agreement with data of Ettenberg and Kressel on variation of carrier lifetime with active layer thickness.^[^ ^26^ ^]^ According to an analysis of AlGaAs/GaAs heterojunction structures, Dupont et al. determined that heterostructure AlGaAs/GaAs LEDs with a thick active layer exhibit high external quantum efficiency (EQE) due to reabsorption in the active layer caused by the PR effect and reported a 22% EQE that corresponds to a 98% internal quantum efficiency (IQE).^[^ ^50^ ^]^

In the aforementioned cases, increasing the active layer thickness promoted PR without degrading the electrical properties of the devices because the active layers were crystalline and, hence, exhibited a long carrier diffusion length. Importantly, it is known that most amorphous or polycrystalline active materials (like amorphous silicon, perovskite and organic polymer semiconductors) have much lower carrier mobilities than crystalline semiconductors. For amorphous or polycrystalline semiconductors, in order to balance between optical absorption and electronic mobility, the films are often kept optically thin which limits PR by the relatively simple approach of increasing the thickness of the active layer. However, thin‐film semiconductor devices have been more popular in view of their great promise for large area coverage, mechanical flexibility and low cost. Therefore, methods to achieve PR in thin‐film devices have also been widely investigated in the last several decades.

### 3.2 Rear‐Reflector Mirrors

The PR effect has been studied in a great number of technological fields because this effect can be maximized through using a rear‐reflector mirror that has a reflectance of >90%. Rear‐reflector mirrors play an important role in reflecting emitted photons back into a semiconductor layer and thereby increasing the probability of PR. Thus, this method has been applied in reflection modulators,^[^ ^51^ ^]^ photodiodes,^[^ ^52^ ^]^ LEDs,^[^ ^53^, ^54^, ^55^, ^56^, ^57^, ^58^ ^]^ and solar cells,^[^ ^59^, ^60^, ^61^, ^62^, ^63^, ^64^, ^65^, ^66^, ^67^, ^68^, ^69^, ^70^, ^71^, ^72^, ^73^ ^]^ because it is a compact and low‐profile solution to improve the efficiency of semiconductor devices. In 1991, Yoffe fabricated high‐efficiency reflection modulators using lift‐off GaAs/AlGaAs epitaxial active layers.^[^ ^51^ ^]^ These layers were bonded to silicon wafers coated with a Au film that served as the rear‐reflector mirror and also as an electrical contact. Although the PR effect was not directly mentioned, this study reported the best results from all epitaxial devices at that time, with modulation ratios up to 40:1 and reflectivity changes over 60%, due to the reflectance from a rear‐reflector mirror. Subsequently, Ho et al. evaluated the effectiveness of both Ti/Pt/Au and Au reflectors for enhancing the photoresponse of InGaAs pin photodiodes. According to their experimental results and theoretical calculations, they concluded the metals with high reflectivity in the infrared range like Au, Ag and Cu are better for use as reflectors to promote the responsivity of vertically illuminated InGaAs pin photodiodes because of the high quantum efficiency of devices obtained compared to those devices with Ti/Pt/Au reflectors.^[^ ^52^ ^]^ In addition, the rear‐reflector mirror system is also popular for use in LED devices. For example, Lee et al. applied a system composed of a 100 nm thick ITO current spreading layer, a 100 nm Al_2_O_3_ low index layer and a 300 nm Al metal layer in the fabrication of AlGaInP LEDs to enhance the output power with only a slightly higher forward voltage.^[^ ^53^ ^]^ The aforementioned examples did not study the PR effect and the main function of the rear‐reflector mirror was to improve light management by either increasing primary absorption of incident photons, or by increasing light‐extraction efficiency. However, rear‐reflector mirrors have the potential to aid in increasing PR because energy transport is not only limited by diffusive charge transport but can occur over long distances through multiple absorption‐diffusion‐emission events.^[^ ^29^ ^]^

**Figure 4:**
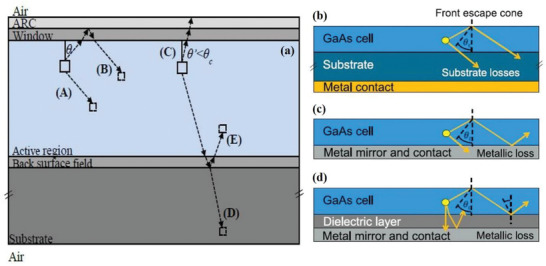
Schematic of optical processes in a single junction solar cell on a substrate. a) Including the PR process (Process A), possible PR processes (Process B and Process E) and processes unrelated with PR (Process C and Process D). The spontaneous emission due to radiative recombination (dashed arrow) occurring at a position within the active layer (full square) can either contribute to a PR generation rate at another position marked by a dashed square (Process A) or after total internal reflection from the top surface (Process B). Another possibility includes a photon flux emitted within the critical angle escaping the cell through the front surface (Process C). Lastly, photons can be emitted downward and lost to the substrate through transmission (Process D). In the case of substrate removal, a back mirror can be placed below the back surface field, thus allowing for reflection and reabsorption in the active layer (Process E). Schematic cross‐section of a solar cell on: b) a dielectric substrate; c) a metallic mirror; and d) with a dielectric layer between the photoactive layer and the metallic mirror. a) Reproduced with permission.^[^ ^60^ ^]^ Copyright 2015, SPIE. b‐d) Reproduced with permission.^[^ ^66^ ^]^ Copyright 2018, IEEE.

Moreover, the use of rear‐reflector mirrors has attracted considerable research interests because they are able to improve the performance of solar cells, especially solar cells using III‐V materials as the active materials. Most III‐V materials used for solar cells, such as GaAs and InGaP, are semiconductors with direct band gaps and high absorption coefficients.^[^ ^59^ ^]^ Hence, radiatively dominated III‐V thin‐film solar cells have shown tremendous potential in exploring the influence of light management techniques to improve device performance. It is known that thinning the thickness of the active material of a solar cell device can reduce the volume within which both radiative and nonradiative recombination occur. In other words, this can reduce the dark current of the cell, and the open‐circuit voltage (*V*_oc_) can be effectively increased.^[^ ^60^ ^]^ However, thinning the active layer of a solar cell can also reduce the photon path length, and, hence, photon absorption, which strongly influences the photocurrent of the cell and the overall PCE. Therefore, devices can benefit from PR effects through highly reflective rear‐reflector mirrors, which uses re‐emitted photons (i.e., secondary photons) that arise from previously absorbed incident solar photons (i.e., primary photons), to allow for a thinning of active material whilst mitigating losses in photocurrent.^[^ ^61^ ^]^ Compared to the PR effect, another method to increase photocurrent is to achieve light trapping/absorption enhancement, which uses incident photons (i.e., primary photons) and ensures that these primary photons are fully reflected back into the device for a second or multiple pass at absorption. Actually, it is important to make a distinction between these two methods because one is the PR effect and the other is absorption enhancement of incident photons through better light trapping. Walker et al. schematically depicted a number of optical processes that occur when electron–hole pairs recombine radiatively at a position within the active layer of a single junction solar cell on a substrate in **Figure** **4**a.^[^ ^60^ ^]^ The first and most basic process includes photons being reabsorbed within the same active layer (Process A). This can also occur via total internal reflection from the top of the cell, and subsequent reabsorption within the same active layer (Process B). Alternatively, the photons emitted toward the top surface can be lost if emitted within the escape cone (Process C). Another possibility is that photons are emitted downward (Process D), and transmitted and lost to the substrate. This loss mechanism can be substantially mitigated by removing the substrate and applying a highly reflective back mirror, such that photons are reflected and reabsorbed in the active layer (Process E). It is obvious that Process A can fully achieve PR in the active layer. In contrast, Process B and Process E may lead to a further exploitation of the PR effect because of the reflected and reabsorbed photons in the active layer.

In the past, the most popular technique to obtain a III–V thin‐film solar cell is the epitaxial lift‐off (ELO) process, in which the cell structure is separated from the substrate by selective wet etching of a thin release layer.^[^ ^62^ ^]^ Bauhuis et al. focused on how to increase the performance of thin‐film GaAs solar cells made through the ELO process during the last decade. In 2009, they reported that thin‐film GaAs solar cells fabricated with a rear‐reflector mirror achieved a record efficiency of 26.1% for a single junction solar cell under 1‐sun illumination (AM 1.5G) conditions due to the PR effect from the reabsorption of low‐energy photons reflected by the mirror layer.^[^ ^62^ ^]^ Furthermore, in order to confine the internal luminescence and exploit PR, Schilling et al. studied the influence of PR under different concentrated illuminations for a GaAs heterojunction solar cell fabricated on a GaAs bulk substrate or on a highly reflecting magnesium fluoride (MgF_2_)/Ag rear‐reflector mirror. They demonstrated that the MgF_2_/Ag mirror can reflect emitted photons back into the absorber layer to lengthen the pathway sufficiently and increase the probability of PR. The cross sections of the GaAs solar cells are schematically shown in Figure 4. In Figure 4b on the rear side, emitted photons with all angles are usually transmitted into the GaAs substrate where parasitic absorption occurs. After replacing the substrate by a mirror, shown in Figure 4c, the reflectivity is generally increased and parasitic absorption loss in the substrate decreases. However, losses can be decreased more by introducing an additional dielectric layer between the photoactive layer and the metal mirror (Figure 4d). In this concept, emitted photons that are transmitted through the dielectric layer have a second chance for reflection at the metallic mirror.^[^ ^66^ ^]^ Based on the research of rear‐reflector mirror systems, Miller et al. considered GaAs thin‐film solar cells with another geometry to explore the physics of light extraction with a randomly textured front surface and a perfectly reflecting mirror on the rear surface.^[^ ^67^ ^]^ The textured surface not only can improve light extraction but it can enhance photon reabsorption, while the rear‐reflector mirror can ensure that the photons can only exit from the front surface not the rear. They reported that GaAs solar cells with this geometry showed the best performance at that time, whose maximum efficiency is 33.5%, due to both PR effect and absorption enhancement.

Recently, a rear‐reflector mirror was used in silicon solar cells^[^ ^68^ ^]^ and perovskite solar cells^[^ ^69^, ^70^, ^71^, ^72^ ^]^ to improve the PCE. For example, Sha et al. demonstrated the efficiency limits of methylammonium lead iodide (CH_3_NH_3_PbI_3_) perovskite solar cells were theoretically and experimentally enhanced by using a rear reflector.^[^ ^70^, ^71^ ^]^ Although these references did not state that PR can influence the performance of solar cells, we expect that their approaches may not only enhance primary absorption through light trapping but also increase the PR. Therefore, the study of mirrors in the solar cells is still an area needs further development because the use of rear‐reflector mirrors is potentially one of the simplest methods to achieve PR effects and it has been comprehensively demonstrated that the mirrors can increase the efficiency of devices.

### 3.3 Photonic Nanostructures

It is known that photonic nanostructures, including optical microcavities, plasmonic nanostructures and photonic crystals, can enhance light harvesting and absorption by manipulating and confining light on the nanometer scale.^[^ ^74^, ^75^, ^76^, ^77^ ^]^ This approach has provided opportunities to improve the efficiency of solar energy conversion in the last decade, especially for optoelectronic devices like solar cells. However, it has been reported that photonic nanostructures also have the potential to achieve PR effects in semiconductor devices.^[^ ^78^ ^]^

#### 3.3.1 Optical Microcavities

In general, optical microcavities are structures that enable the confinement of light to microscale volumes. The universal importance of these structures has made them indispensable in a wide range of fields.^[^ ^78^ ^]^ On the other hand, optical microcavities can also be used to achieve PR in semiconductor devices because the spontaneous emission rate can be modified with cavities due to the larger or smaller density of optical states in the cavity as compared with the free space.^[^ ^79^ ^]^ In 1997, Raj et al. theoretically approximated the PR effect in semiconductor lasers with quantum structures and reported that the threshold current reduced due to the PR effect in compressively low‐dimensional quantum well structures.^[^ ^80^ ^]^ Furthermore, as discussed earlier, Numai calculated PR efficiencies for circular, square, and triangular ring cavities in semiconductor lasers in 2000.^[^ ^48^ ^]^ In 2012, Liu et al. investigated the effect of microcavity size and density on the PCE of dye‐sensitized solar cells (DSSCs).^[^ ^81^ ^]^ In this case, the increase in optical absorption and incident monochromatic PCE in the long‐wavelength region indicated that the enhancement of cell performance with the increase of both *V*_oc_ and short‐circuit current density (*J*_sc_) was due to PR as well as the multiple scattering of light by the microcavities and light confinement by the stack of TiO_2_ layers.

#### 3.3.2 Plasmonic Nanostructures

The ability of noble metal nanostructures to manipulate light at the nanoscale resulted in the research area of plasmonics.^[^ ^82^ ^]^ Plasmonics is one possible technology that can enable electronic and photonic components to be combined on the same chip.^[^ ^83^, ^84^, ^85^ ^]^ The incorporation of metal nanostructures into optoelectronic devices has been extensively studied over the past decade as it is believed to be a promising approach to enhance device performance without increasing the size of devices.^[^ ^85^ ^]^ Nowadays, plasmonic structures offer at least three ways of reducing the physical thickness of photovoltaic (PV) absorber layers while keeping their optical thickness constant. Briefly, these three plasmonic effects include plasmonic light scattering, localized surface plasmon resonances (LSPRs) and surface plasmon polaritons (SPPs).^[^ ^86^ ^]^ There is no doubt that plasmonic nanostructures can improve the performance of solar cells through enhancing light trapping to increase the photon absorption.^[^ ^83^, ^86^ ^]^ However, PR effects can also be induced by plasmonic nanostructures to enhance photon reabsorption and increase the *V*_oc_ of solar cells. Saliba et al. have reported that core‐shell silver‐titania nanoparticles (Ag@TiO_2_) were successfully incorporated in organic‐inorganic perovskite solar cells through a low‐temperature processing method.^[^ ^87^ ^]^ In this case, a significant enhancement in the performance of devices was observed, delivering a PCE of 16.3% with 0.03 V increase of *V*_oc_ through optimizing the concentration of Ag@TiO_2_ in the active layer of the device. They explained that photons emitted from exciton recombination would have an extended optical pathlength because they interact with the highly polarizable Ag nanoparticles (AgNPs) that are lying in the plane of the perovskite film. Therefore, the highly polarizable AgNPs act as antennas for light re‐emitted from radiative recombination of electron‐hole pairs and the re‐emitted light can be reabsorbed by the perovskite film again.^[^ ^87^ ^]^ Since this is the only work that mentions PR in solar cells achieved by using plasmonic nanostructures, knowledge is lacking on how much impact plasmonic nanostructures can have on PR in solar cells. Therefore, this is an aspect that needs further study in the future.

### 3.4 Combination of the Above Methods–Nanostructured Rear‐Reflector Mirror

Although a vast amount of research has focused on photonic nanostructures, which strongly enhance the interaction of light with nanoscale matter, some photonic nanostructures made from metals suffer from strong parasitic absorption losses in metals at optical frequencies, thereby hampering the performance of most of their potential applications.^[^ ^88^, ^89^, ^90^ ^]^ Usually, metal nanoparticles can improve absorption via plasmonic resonances, but their beneficial effects are countered by metal absorption losses at short wavelengths. In order to minimize the disadvantages associated with use of metal nanoparticles, they could be integrated with reflective back mirrors to aid in light‐trapping and photon reabsorption.^[^ ^88^, ^91^ ^]^ Additionally, rear‐reflectors can significantly increase the value of the *V*_oc_ of devices compared to the control group, to improve the PCE of the devices by the PR effect^[^ ^67^, ^92^, ^93^, ^94^, ^95^ ^]^ through back reflections discussed in Section 3.2. Therefore, combining nanostructures and rear‐reflector mirrors together to obtain nanostructured rear‐reflector mirrors may be used to increase long‐wavelength reabsorption and improve device performance.

**Figure 5:**
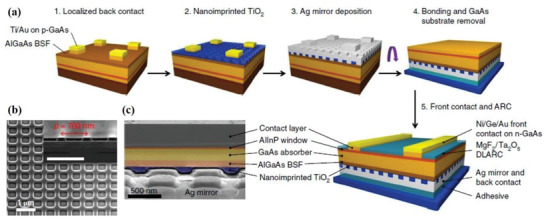
Fabrication process for ultrathin GaAs solar cells with a nanostructured rear‐reflector mirror. a) Sketches of the main fabrication steps. b) SEM image of nanoimprinted TiO_2_ periodic structures before Ag back mirror deposition. c) SEM cross‐sectional view after removing the GaAs substrate, showing the nanostructured rear‐reflector mirror. a–c) Reproduced with permission.^[^ ^94^ ^]^ Copyright 2019, Nature Publishing Group.

Recently, Chen et al. proposed a strategy based on multi‐resonant absorption in a planar active layer, which used a nanostructured Ag rear‐reflector mirror fabricated by soft nanoimprint lithography.^[^ ^94^ ^]^ **Figure** **5**a schematically shows the solar cell fabrication process sequence with a nanostructured Ag rear‐reflector mirror. Figure 5b,c show scanning electron microscopy (SEM) images of a nanoimprinted two‐dimensional (2D) periodic TiO_2_ grating and a cross‐sectional SEM image of a TiO_2_/Ag rear‐reflector mirror, respectively. In this work, a certified PCE of 19.9% was demonstrated using a 205‐nm‐thick GaAs active layer and a Ag nanostructured rear‐reflector mirror with a periodic pattern. Compared to the reference cells of regular thin‐film single junction GaAs solar cells, the value of *V*_oc_ increased by around 0.1 V using the nanostructured rear‐reflector mirror for a device with similar thickness of the active material,^[^ ^94^ ^]^ which is attributed to the repeated recycling between photons and electron‐hole pairs that can create high excitation densities and allow high *V*_oc_.

## 4 Characterization of Photon Recycling

From the examples discussed in Sections 1, 2, 3 above, it is apparent that the PR can be achieved in a great number of semiconductors to improve device performance, such as lasers, LEDs, and solar cells. However, there is no doubt that a lot of different characteristics are used to quantify performance and characterize properties of different semiconductor devices. Thus, we describe some ways in which PR can be characterized and distinguished from other light‐matter interactions. Additionally, we review how PR affects device characteristics in different optoelectronic applications. **Table** **2** summarizes potential methods to characterize or identify PR in different sample types and relates the methods to applications. According to the Table 2, edge emission measurements are applicable to almost all applications and the existence of PR can be confirmed through the change of edge emission intensity at short wavelengths. This is one of the most direct methods for characterizing PR, so far. In contrast to edge emission measurements, the other PR characterization methods are specific to the particular application; for example, output power and light extraction efficiency (LEE) are specific characteristics for lasers and LEDs, respectively. These are more indirect ways of characterizing PR as they can be convoluted by other photonic effects. Therefore, for light emitting devices, methods for the characterization of PR still need further development. If the impact of PR on device performance can be readily quantified in practice, PR may play a more accurate and targeted role in the improvement of the performance of optoelectronic devices.

**Table 2:** Summary of different methods to characterize PR in different applications

Characterization	Active materials	Sample types	Application	Reference no.
Edge emission measurements	PFO	Film	Nanoscale waveguide	^[^ ^101^ ^]^
	CdS_x_Se_1‐x_	Nanowire	Nanoscale waveguide	^[^ ^102^ ^]^
	CdS	Nanowire	Nanoscale waveguide	^[^ ^102^ ^]^
	CdS_x_Se_1‐x_	Nanowire	Laser	^[^ ^103^ ^]^
	CdS/CdS_x_Se_1‐x_	Nanowire	Photodetector	^[^ ^104^ ^]^
	CdSe	Nanowire	Nanoscale waveguide	^[^ ^105^ ^]^
	GaN/InGaN	Microrod	Nanoscale waveguide	^[^ ^106^ ^]^
	GaAs	Nanowire	Solar cell	^[^ ^108^ ^]^
	DMHP	Nanowire	Laser	^[^ ^109^ ^]^
	Perovskite	Film	Solar cell	^[^ ^29^ ^]^
Output power	AlGaAs/GaAs	Device	Laser	^[^ ^115^ ^]^
LEE	GaN	Device	LED	^[^ ^119^ ^]^
TRPL	Perovskite	Film	Solar cell	^[^ ^28^ ^]^
	Perovskite	Film	Semiconductor	^[^ ^120^ ^]^
	Perovskite	Film	LED	^[^ ^121^ ^]^
*I* *–V*	Perovskite	Device	Solar cell	^[^ ^87^ ^]^

**Note**: DMHP = (*E*)‐3‐(4‐(dimethylamino)‐2‐methoxyphenyl)‐1‐(1‐hydroxynaphthalen‐2‐yl)prop‐2‐en‐1‐one.

### 4.1 Edge Emission fromSemiconductor Waveguides

**Figure 6:**
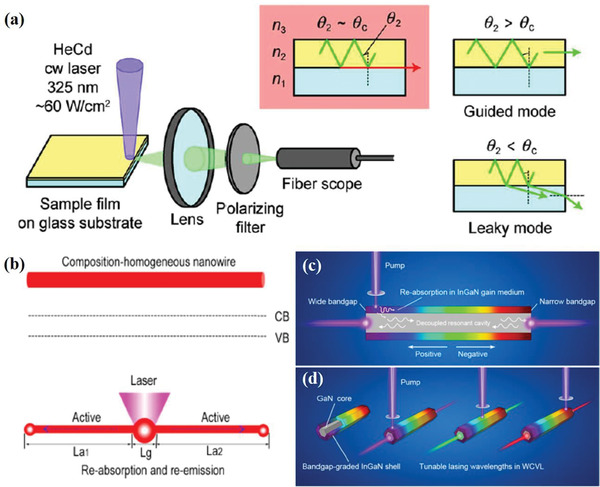
a) Schematic illustration of experimental setup of cutoff emission measurement for detection of anisotropy in organic amorphous films. Organic films on glass substrates were optically pumped near the edges of the substrates by ultraviolet light from a continuous wave He–Cd laser. Spectral shapes of edge emissions often significantly differ from the normal PL spectra shapes, and the peak wavelengths of the narrowed bands are in good agreement with the cutoff wavelengths of the slab waveguides composed of the glass substrate (with a refractive index of *n*_1_), organic film (*n*_2_) and air (*n*_3_). b) Schematic diagram of a composition‐homogeneous nanowire, its band gap structure along the axial direction and schematic waveguiding with local excitation at the center of the nanowire. The emitted light is actively guided toward the two ends through reabsorption and re‐emission (Lg: Gain length; La_1_, La_2_: Absorption length). c) Schematic diagram of the structure that decouples the gain medium and resonant cavity. d) Effect diagram of WCVLs, which can be obtained by simply changing the excited position without additional operation. a) Reproduced with permission.^[^ ^96^ ^]^ Copyright 2009, Elsevier. b) Reproduced with permission.^[^ ^103^ ^]^ Copyright 2013, American Chemical Society. c,d) Reproduced with permission.^[^ ^106^ ^]^ Copyright 2017, American Chemical Society.

Edge emission measurements, also known as cutoff emission measurements, are used to study a boundary phenomenon between the cases where light is guided and where light is leaky, which means that the incident angle in a thin film *θ*_2_ corresponds to the critical angle of the waveguide *θ*_c_ (**Figure** **6**a).^[^ ^96^, ^97^, ^98^, ^99^, ^100^, ^101^ ^]^ Edge emission measurements are also capable of detecting guided modes (i.e., when *θ*_2_ > *θ*_c_). At the cutoff wavelength for the waveguide, the angle for propagation will be equal to the critical angle for the active material/glass interface and no total internal reflection will occur. As a result, some of the light will be lost from the guided mode and some will propagate in the substrate parallel to the plane of the film.^[^ ^92^ ^]^ In the past decades, edge emission measurements were important methods to study amplified spontaneous emission (ASE) in high‐gain conjugated polymer films.^[^ ^97^, ^98^, ^99^, ^100^, ^101^ ^]^ In these studies, photons emitted inside the polymer films are guided to the sample edge. Edge emission measurements have the potential to be used to characterize PR because of the collection of emission that is trapped in propagating modes. The change in the spectral shape of the emission collected at the sample edge can be analyzed to determine if emitted photons were reabsorbed at wavelengths near the band gap of the semiconductor film.

Dalsania et al. studied exciton emitter‐SPP coupling in a plasmonic waveguide configuration and investigated the effects of changing metal film thickness as well as gain in the semiconducting polymer on the coupled emission based on the data from edge emission measurements.^[^ ^101^ ^]^ In this case, edge emission spectra from insulator‐semiconductor‐metal‐insulator (ISMI) waveguides were collected over a range of optical excitation pump power densities and unpolarized emission spectra as well as both transverse electric (TE)‐ and transverse magnetic (TM)‐polarized emission spectra were probed. It was found that the edge emission behavior from the waveguides depended on both the Ag film thickness and roughness.^[^ ^101^ ^]^ The shape of the emission spectra showed clear signatures of reabsorption of shorter wavelength emission similar to what occurs in PR.

Edge emission measurements have also been widely used to study reabsorption and PR in nanowires. Under laser excitation, almost all parts of the emitted photons from a semiconductor nanowire, such as CdS_x_Se_1‐__x_,^[^ ^102^, ^103^, ^104^, ^105^ ^]^ GaN,^[^ ^106^, ^107^ ^]^ GaAs,^[^ ^108^ ^]^ and single‐crystalline organic nanowire,^[^ ^109^ ^]^ can be reabsorbed by other parts of the nanowire to achieve PR.^[^ ^102^, ^103^, ^104^, ^105^, ^106^, ^107^, ^108^, ^109^, ^110^ ^]^ Pan et al. reported that the excited light can be actively guided toward the two opposite ends of a nanowire through reabsorption and re‐emission processes when a focused laser beam excites the nanowire near its center (Figure 6b).^[^ ^102^, ^103^ ^]^ Furthermore, Zong et al. proposed a single GaN/InGaN core‐shell microrod with a length of 10 µm to obtain high‐performance, low‐cost, wavelength continuously variable lasers (WCVLs) by decoupling a gain medium and resonant cavity.^[^ ^106^ ^]^ In this core‐shell structure, the band gap‐graded InGaN shell provides the gain medium, while the high‐quality GaN core acts as the resonant cavity. When the wide band gap end of the shell layer is locally excited, the residual light in the shell layer will transmit along the negative direction through repeated band‐to‐band reabsorption and re‐emitting processes (Figure 6c).^[^ ^106^ ^]^ Once the size parameters of core‐shell structures are designed and optimized, the majority of optical field intensity will be distributed in the core, which means the gain medium and resonant cavity can sufficiently decouple. In this case, energy from excitation by an external ultraviolet laser spot can be absorbed strongly and locally at the excited position with a certain indium composition, and then the emission with the corresponding wavelength can oscillate in the core without mutual absorption. Therefore, a tunable laser can be obtained through simply changing the excitation position (Figure 6d).

**Figure 7:**
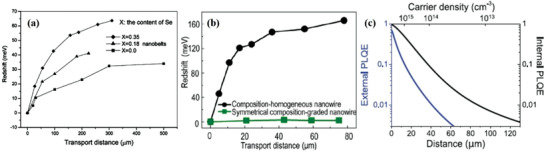
a) The plots of redshift value in photon energy versus excitation position (transport distance) for CdS_0.65_Se_0.35_, Cds_0.82_Se_0.18_, and CdS nanobelts, respectively. b) Propagation distance‐dependent spectral redshift of the detected output signals at the end of the wire. The propagation distance is calculated from the excitation spot to the wire end. c) Change in external and internal PLQE as a function of distance derived from a PR model. a) Reproduced with permission.^[^ ^102^ ^]^ Copyright 2007, American Chemical Society. b) Reproduced with permission.^[^ ^103^ ^]^ Copyright 2013, American Chemical Society. c) Reproduced with permission.^[^ ^29^ ^]^ Copyright 2016, American Association for the Advancement of Science.

Propagation distance‐dependent PL spectra plays an important role in detecting output signals at the edge or the end of materials and are useful when characterizing reabsorption and PR. For example, **Figure** **7**a,b show the propagation distance‐dependent spectral redshift plots of three nanobelts with different compositions (CdS_0.65_Se_0.35_, CdS_0.82_Se_0.18_ and CdS)^[^ ^102^ ^]^ and redshift comparisons between a composition‐homogeneous nanowire and a symmetrical composition‐graded nanowire,^[^ ^103^ ^]^ respectively. It is apparent that as the propagation distance increases, the redshift of the emission spectrum increases, which arises from increased reabsorption in the emitting material.

Similarly, Pazos–Outón et al. studied PR effects in lead iodide perovskite solar cells by carrying out PL quantum yield (PLQE) measurements as a function of distance from the excitation spot.^[^ ^29^, ^111^ ^]^ The PLQE of a molecule or material is defined as the number of photons emitted divided by the number of photons absorbed. It is one of the most important characteristics in thin films because it is able to give reliable determination of PL efficiency.^[^ ^112^ ^]^ In Figure 7c, Pazos‐Outón et al. showed that both external PLQE and internal PLQE vary with distance from the excitation spot.^[^ ^29^ ^]^ The carrier density determined by theoretical calculations was also indicated and decreased with increasing distance from the excitation spot. As a result, it was determined that PR occurs and it can be efficient near the excitation spot but drops off at larger distances for which charge‐carrier densities are smaller. The reason is that the external PLQE results from multiple internal recycling events and is related to the internal PLQE when the edge emission is measured from one edge of the film to the other to provide a direct probe of the internal photon distribution traveling through the film.^[^ ^29^ ^]^

### 4.2 Output Power of Laser Diodes

**Figure 8:**
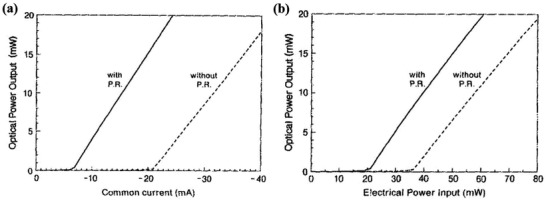
Optical output power for laser diodes as a function of a) the common drive current with and without the use of PR and b) the electrical input power with and without PR. a,b) Reproduced with permission.^[^ ^115^ ^]^ Copyright 1990, AIP Publishing.

It is known that the most fundamental characteristic for checking the performance of a laser is optical output power, which shows the power or energy output of the laser as a function of input power, current or excitation energy. In the past, this characteristic has been studied theoretically and experimentally in the broad field of laser diodes to determine the threshold for lasing.^[^ ^113^, ^114^, ^115^ ^]^ In addition, it also plays an important role in showing properties of laser diodes with and without PR effects. In 1990, Gigase et al. reported that the threshold pump power of an AlGaAs‐GaAs ridge quantum well laser diode can decrease by PR.^[^ ^115^ ^]^ **Figure** **8**a,b show the optical output power as a function of the common drive current and as a function of the electrical input power with and without PR, respectively. In Figure 8a, it is obvious that the threshold current of the laser diode is 20.9 mA without PR and the differential efficiency is 0.77 W A^−1^ in this condition. However, the threshold current of the laser diode with PR through the common contact is just 6.9 mA, which is a reduction of 67% compared to the threshold current when no PR occurred. Additionally, according to the Figure 8b, the total electrical input power at threshold is 36.1 mW without PR, but it has been reduced to 20.9 mW when PR occurs. As a result, the threshold pump power of the AlGaAs‐GaAs ridge quantum well laser diode was reduced by 42% through recycling the spontaneous emission. In other words, PR of optical output power results in a reduction of the electrical input power required to reach the lasing threshold.^[^ ^115^ ^]^

### 4.3 Light Extraction Efficiency of LEDs

LEE is one of the key characteristics to understand the performance of LED devices whose EQE is not high, like GaN‐based LEDs, because the EQE is equal to the multiplication of the IQE and the LEE.^[^ ^116^, ^117^, ^118^, ^119^ ^]^ In 2011, Sun et al. presented a Monte Carlo ray tracing study of light extraction of GaN‐based LEDs through cavity (passive) and quantum (active) PR to improve the performance by enhancing the LEE.^[^ ^119^ ^]^ They discussed the mechanism of the enhancement of light extraction for devices incorporating an implanting lens array and lens encapsulation. Then, they described the mechanism of PR for light extraction enhancement in an LED die. The reflected photons can be absorbed by the active layer and be partially re‐emitted so that the new emitted photons may escape because of the different propagation direction from the original one before absorption. Additionally, they defined a factor of PR rate (*P*_RR_), which can be used to describe the re‐emission rate of an absorbed photon, to understand that PR enhances light extraction more dominantly in an LED die with a larger absorption coefficient in the active layer and with a larger *P*_RR_. Thus, they obtained the relationship between LEE and PR rate by using different absorption coefficients (10 000 and 200 cm^−1^) because the absorption coefficient is the major constraint to light extraction in the active layer of LEDs. In conclusion, they demonstrated that both approaches (implanting lens array and lens encapsulation) perform more than 90% of LEE through cavity PR for an absorption coefficient of 200 cm^−1^ in the active layer. For a heavily absorbing active layer with an absorption coefficient of 10 000 cm^−1^, quantum PR can play an important role in enhancing the LEE of LEDs.^[^ ^119^ ^]^ Generally, cavity PR in LED dies is one of the key approaches to enhance light extraction because an LED die can be regarded as a rectangular cavity where photons are emitted from the active layer and are incident on all exit surfaces to escape the cavity. Therefore, an effective cavity of the LED die is able to increase the possibility of photons to be reabsorbed by changing the propagation direction. On the other hand, quantum PR is described as the case where reflected photons can be absorbed by the active layer and partially be re‐emitted so that the new emission photons may escape the LED die because of the change of the propagation direction.

### 4.4 Time‐Resolved Photoluminescence of Solar Cells

Time‐resolved PL (TRPL) is an experimental technique that provides the spectral and temporal evolution of the emission of a sample following its illumination by a short pulse of light. The short pulse of light generates electron–hole pairs that decay to lower energy excited states of the sample; then, these electron‐hole pairs can recombine and emit light. The emitted light is composed of a set of wavelengths corresponding to transition energies, so the measurement of the optical spectrum as a function of time provides a means to measure the transition energies and their lifetimes. Recently, this measurement has become an important method to study PR in semiconductor devices due to the multiple cycles of regeneration of charge carriers through self‐absorption.^[^ ^28^, ^120^, ^121^ ^]^

**Figure 9:**
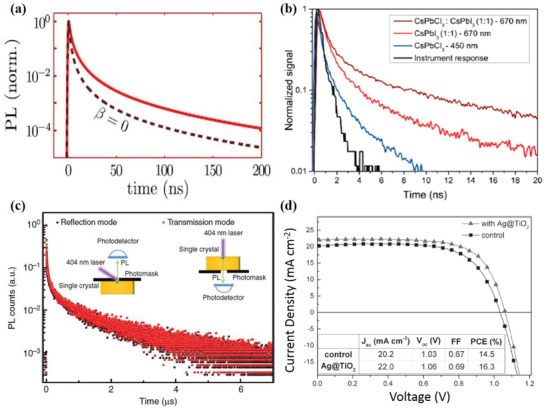
PL lifetime decays of: a) a metal halide perovskite thin‐film semiconductor with (red full line) and without (black dash line) PR (simulated data); b) different perovskite nanocrystals excited at 405 nm with measurements at 450 or 670 nm; and c) a CH_3_NH_3_PbBr_3_ single crystal. d) *J*–*V* curves for perovskite solar cells for both a control device and an optimized Ag@TiO_2_ device. a) Reproduced with permission.^[^ ^120^ ^]^ Copyright 2018, American Physical Society. b) Reproduced with permission.^[^ ^121^ ^]^ Copyright 2017, American Chemical Society. c) Reproduced with permission.^[^ ^28^ ^]^ Copyright 2017, Nature Publishing Group. d) Reproduced with permission.^[^ ^87^ ^]^ Copyright 2015, Wiley‐VCH.

PR has been shown to improve the performance of perovskite solar cells because of the efficient radiative recombination. For example, Ansari‐Rad and Bisquert plotted PL lifetime decays of perovskite thin‐film semiconductors with and without considering the PR at high excitation fluence (10^14^ photons cm^−2^) from simulations that modeled the kinetics of the secondary photons in the film as a diffusion process with appropriate creation and annihilation terms (**Figure** **9**a).^[^ ^120^ ^]^ It is apparent that the PL lifetime decay is faster without PR by imposing *β* = 0, which is the photon absorption rate constant that is the inverse of the lifetime of the photon, because photocarriers lost by radiative recombination do not have any chance to be regenerated. Davis et al. also reported that the CsPbCl_3_ emission can be reabsorbed by CsPbI_3_ nanocrystals because of the large absorption coefficient of the CsPbI_3_ nanocrystals in the range of the CsPbCl_3_ emission through the study of interactions in blend films with mixtures of different CsPbX_3_ (X = Cl, I) perovskite nanocrystals.^[^ ^121^ ^]^ The existence of PR in a mixture of CsPbCl_3_ and CsPbI_3_ perovskite nanocrystals with different crystal compositions has been demonstrated. In Figure 9b, for mixed samples with a 1:1 ratio of CsPbCl_3_:CsPbI_3_ by weight, the nanocrystals showed an increased lifetime in TRPL when excited at 405 nm with the measurement at 670 nm compared to CsPbI_3_ nanocrystals. Some emission was also detected from CsPbCl_3_ nanocrystals measured at 450 nm but no emission was detected at a wavelength of 670 nm. Therefore, Davis et al. ascribed the dominant emission from CsPbI_3_ in the CsPbCl_3_:CsPbI_3_ nanocrystals to efficient reabsorption of photons emitted from CsPbCl_3_ nanocrystals.^[^ ^121^ ^]^ On the other hand, Fang et al. confirmed that PR is not significant in their perovskite single crystals through TRPL decay curves measured in both transmission and reflection modes (Figure 9c).^[^ ^28^ ^]^

### 4.5 Current–Voltage Characteristics of Solar Cells

It is known that PR plays an important role in solar cells to improve the performance of devices. In this regard, current‐voltage (*I*–*V*) characteristics under solar illumination are one of the primary indicators of PR in solar cells because *V*_oc_, which is affected by PR, can be readily determined from them. Saliba et al. indicated that plasmonic‐induced PR may help to move highly efficient perovskite solar cells closer to the theoretical limiting efficiencies.^[^ ^87^ ^]^ They showed that core‐shell Ag@TiO_2_ nanoparticles can be incorporated into perovskite solar cells through a low‐temperature processing route to boost the measured PCE. According to the *J*–*V* curves in Figure 9d, the best control and Ag@TiO_2_ devices exhibited PCEs of 14.5% and 16.3%, respectively. Furthermore, the average PCE increased by ~20% from 11.4% ± 1.3% to 13.7% ± 1.2%. Although the enhancement is mainly due to increased *J*_sc_, the isolated perovskite films exhibit a quenching of the externally measured PL which would generally indicate an increase in disadvantageous nonradiative decay pathways in the solar cells.^[^ ^87^ ^]^ Therefore, the increase of the *V*_oc_ due to PR also contributed to the increase in the PCE of the solar cells in this case.

## 5 Photon Recycling in Optoelectronic Applications

PR has been achieved in a wide range of applications to improve performance. In this section, we review optoelectronic applications of the PR effect, including semiconductor lasers, LEDs, and solar cells. In the past, reabsorption was typically regarded as a loss mechanism in LEDs; however, more recently, reabsorption and the resulting PR has been reported to play a positive role in LEDs. In that regard, PR can assist with optical coupling through randomizing the direction of photon propagation and redirecting photons from trapped to outcoupled modes. While PR has been used to improve the performance of light‐emitting devices (LEDs, lasers), it is most widely studied in solar cell applications. In contrast to lasers and LEDs, PR has more obvious benefits for the performance of solar cells because solar cells have a greater demand for photon absorption than lasers and LEDs, and PR can significantly promote and meet this requirement. According to the reported work so far, PR can affect the efficiency of solar cells by enhancing the *V* *_oc_* by factors of up to 1.24 in theory and 1.08 in experiment, but in many cases it has only a small effect. Although PR has not been reported to dramatically increase the efficiency of solar cells to date, controlling PR can still lead to breakthroughs in the performance of devices already close to the value of Shockley‐Queisser (SQ) limit. In addition, PR has not been investigated and controlled well in organic semiconductor materials and devices. Therefore, there are still many opportunities for research on the role of PR in semiconductor materials and devices that are still under development.

### 5.1 Photon Recycling in Semiconductor Lasers

**Figure 10:**
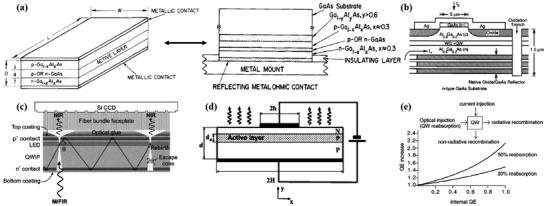
a) Schematic diagram of laser structure and cross‐sectional view of an intermediate stage in a feasible processing scheme for fabricating PR lasers. b) Schematic cross section of a native‐oxide vertically confined laser diode. c) Schematic of QWIP‐LED layer structure showing NIR emission and absorption/re‐emission events. The top coating is antireflective for NIR and reflective for middle/far infrared (M/FIR). The bottom coating is antireflective for M/FIR and reflective for NIR. d) Schematic diagram of the double heterostructure LED in which a narrow‐gap p‐type active layer is sandwiched between two confining n‐ and p‐layers. The size of the n‐type contact (2h) is much smaller than the lateral size of the active layer (2H). The p side is covered by a metal layer serving as a mirror in the LED. e) Increase of the EQE of LEDs due to reabsorption and recycling of spontaneous emission by the active layer. a) Reproduced with permission.^[^ ^122^ ^]^ Copyright 1974, AIP Publishing. b) Reproduced with permission.^[^ ^123^ ^]^ Copyright 1995, AIP Publishing. c) Reproduced with permission.^[^ ^126^ ^]^ Copyright 2000, AIP Publishing. d) Reproduced with permission.^[^ ^128^ ^]^ Copyright 2000, AIP Publishing. e) Reproduced with permission.^[^ ^129^ ^]^ Copyright 1997, AIP Publishing.

Semiconductor lasers are lasers based on semiconductor gain media, where optical gain is usually achieved by stimulated emission from an interband electronic transition under conditions of a high carrier density in the conduction band. In 1974, Stern and Woodall first reported that a suitably designed laser structure can increase the fraction of spontaneous emission that is reabsorbed in the active layer and can decrease the current density that must be supplied to reach the lasing threshold through PR in a GaAs double‐heterostructure semiconductor laser.^[^ ^122^ ^]^ In order to maximize the PR effect in this case, the structures shown schematically in Figure 9a were used, in which only the GaAs layer is the active layer and Ga_1‐__x_Al_x_As films are added as the confining layers. Usually, spontaneously emitted photons can be removed from the system in several ways: (1) they can be emitted from the side and end faces; (2) they can be absorbed in the contacts; (3) they can be absorbed in the bulk by loss processes; and (4) they can be reabsorbed in the active layer. Therefore, Stern and Woodall established the fraction of spontaneously emitted photons of photon energy *E* which is reabsorbed in the active layer is expressed as:^[^ ^122^ ^]^

(15) $$\theta \left(E\right)=\alpha_{i}\left(E\right)d_{l}L_{l}W_{l}{\left[\alpha_{i}\left(E\right)d_{l}L_{l}W_{l}+\alpha_{i}\left(E\right)D_{l}L_{l}W_{l}+\frac{1}{4}T_{d}\left(2D_{l}L_{l}+2D_{l}W_{l}\right)+\frac{1}{4}A_{d}\left(2L_{l}W_{l}\right)\right]}^{-1}$$

where *α*_*i*_(*E*) is the interband absorption coefficient at photon energy *E*, *L*_l_, *W*_l_, and *D*_l_ are the length, the width and the height of the laser (corresponding to L, W and D in **Figure** **10**a, respectively); *d*_l_ is the thickness of the active layer (GaAs) in the laser (corresponding to d in Figure 10a). *T*_d_ and *A*_d_ are the transmissivity and the absorptivity for diffuse light, respectively. Furthermore, the fraction (*θ*_av_) of all the spontaneous emitted photons which is reabsorbed in the active layer is further expressed by Stern and Woodall as:^[^ ^122^ ^]^

(16) $$\theta_{\mathrm{av}}=\left[\int \theta \left(E\right)\gamma_{\mathrm{spon}}\left(E\right)dE\right]{\left[\int \gamma_{\mathrm{spon}}\left(E\right)dE\right]}^{-1}$$

*γ*_spon_ is the spontaneous emission rate at photon energy *E*. It is known that the effective current density (*J* *_eff_*)

(17) $$J_{\mathrm{eff}}=J_{\mathrm{ext}}+\eta_{\mathrm{sp}}\theta_{\mathrm{av}}J_{\mathrm{eff}}$$

required to reach a given excitation level is the sum of the externally supplied current density *J*_ext_ and a current density *η*_sp_ *θ*_av_ *J*_eff_ associated with reabsorption, where *η*_sp_ is the quantum efficiency for spontaneous emission. Therefore, the external current density required to reach threshold is reduced by a fraction *η*_sp_ *θ*_av_.^[^ ^122^ ^]^

The magnitude of the threshold reduction due to PR was estimated to be about 20% in a GaAs double‐heterostructure laser at room temperature.^[^ ^122^ ^]^ In another case, Chen et al. demonstrated an edge‐emitting laser diode of Al_y_Ga_1‐__y_As‐GaAs‐In_x_Ga_1‐__x_As quantum well heterostructures modified by the formation of a distributed Bragg reflecting (DBR) mirror (composed of alternating layers of AlAs‐oxide/GaAs), which added vertical confinement to the longitudinal laser cavity in order to achieve PR (Figure 9b). For these laser diodes, the bottom DBR mirror, combined with the highly reflective top p‐contact metallization (Ag), formed a thin broadband vertical cavity. As a result, the auxiliary vertical mirrors are tuned to improve the coupling of the spontaneous emission to the longitudinal lasing mode, resulting in reduced threshold currents and modified emission characteristics below threshold.^[^ ^123^ ^]^ In addition, as already discussed in Section 4.1, PR plays an important role in modulating lasing wavelength in semiconductor nanowires that have potential applications as micro/nanoscale lasers in integrated nanophotonics.^[^ ^103^, ^106^, ^109^ ^]^ Besides the aforementioned examples, the study of PR in lasers is relatively limited despite the potential benefits in controlling spontaneous emission.

### 5.2 Photon Recycling in Light‐Emitting Diodes

Although reabsorption is typically considered to be a loss mechanism in LEDs,^[^ ^124^, ^125^ ^]^ the effect of PR associated with the reabsorption of nonextracted light in LEDs is still important for improving performance. This is because PR can significantly assist with optical coupling by randomizing the direction of photon propagation and redirecting photons from trapped to outcoupled modes to enhance the EQE of LED devices. The EQE enhancement of LED devices is achieved by the re‐emission of photons generated in the active layer with directions outside the critical cone of angles.^[^ ^126^, ^127^, ^128^, ^129^ ^]^ Dupant and Chiu theoretically studied the major device parameters influencing the near infrared (NIR) light lateral spread and the LED external efficiency of a quantum well infrared photodetector (QWIP) integrated with a LED (QWIP‐LED) imaging device (Figure 9c). More specifically, they studied structures coupling a QWIP and a LED that use PR effects achieved by optimizing the thickness of the LED active layer (600 nm) for high EQEs.^[^ ^126^ ^]^ According to the present PR LED model proposed by Schnitzer et al.,^[^ ^11^, ^127^ ^]^ the final expression for the QWIP‐LED EQE, *η*_ext_, was given by

(18) $$\eta_{\mathrm{ext}}=p_{e}\eta_{\mathrm{int}}+\;\frac{p_{\mathrm{tr}}p_{e}\eta_{\mathrm{int}}^{2}\alpha_{0}d_{0}}{\left(1-\eta_{\mathrm{int}}p_{\mathrm{tr}}\right)\alpha_{0}d_{0}+\alpha_{0}^{\prime }d_{0}^{\prime }-\frac{\log\left[\left(1-A^{\prime }\right)\left(1-A\right)\right]}{2/\sin\theta '}}$$

where *p*_e_ is the average escape probability of the light inside both the forward and backward cones, *η*_int_ is the IQE of the LED, *p*_tr_ is the average probability that photons stay trapped in the cavity after isotropic LED emission (can be approximated by 1 − *p*_e_), *α*_0_ *d*_0_ (*α*_0_ ≈ 5000 cm^−1^ is the wavenumber and *d*_0_ is the thickness of the LED active layer) is the absorption of the LED active layer, $\alpha_{0}^{\prime }d_{0}^{\prime }$ ($\alpha_{0}^{\prime }$ is the wavenumber and $d_{0}^{\prime }$ is the thickness of the other layers of the LED) is the parasitic absorption in all layers (mainly the doped contact layers) except the LED active layer, *θ'* is an average angle that all the photons depart (see Figure 9c), *A*′ and *A* are the absorption coefficient of the top coating and bottom coating, respectively.^[^ ^126^ ^]^ In fact, *p*_e_ increases when PR exists and increases because a large value of *p*_e_ means that more photons can escape from the active layer to enhance EQE. Finally, Dupant and Chiu found that the thickness of the LED is a very important parameter that needs to be increased in order to maximize the EQE.

Furthermore, Tsutsui et al. also studied the effect of PR in theory and calculated the EQE as a function of device parameters in double heterostructure LEDs with relatively small area contacts providing nonuniform injection of electrons (Figure 9d). In that work, the equation of EQE (*η*_ext_) is expressed as

(19) $$\eta_{\mathrm{ext}}=\frac{T_{0}\left(1-\delta \right)\delta F_{\left(\alpha_{a}d_{a}\right)}F_{\left(\alpha^{*}h^{\prime }\right)}}{\left[1-\delta \left(\frac{\alpha '\tau_{l}}{\alpha^{*}\tau_{r}}\right)\right]}\cdot \left(\frac{\alpha '\tau_{l}}{\alpha^{*}\tau_{r}}\right)\left(\frac{\tau_{l}}{\tau_{r}}\right)$$

where *T*_0_ is the transmission coefficient of the outer interface, *δ* is the spontaneous emission factor, *α*'= *α*_a_Γ, α* = *α*_a_Γ + *α*_c_(1 − Γ), *α*_a_ is the absorption coefficient averaged over the emitted photon spectrum, *α*_c_ is the averaged absorption coefficient for the confining layers, Γ is the confinement factor, *τ*_l_ is the net lifetime of electrons in the LED active layer, *τ*_r_ is the radiative recombination lifetime of electrons, which is the same nomenclature in Equation (8), *F* is a mathematical function that is *F*_(*m*)_ = [1 − exp (−2*m*)]/*m*, *d*_a_ is the thickness of the active layer, and 2*h*′ is the width of the n‐type contact in Figure 9d. In Equation (19), the value of *η*_ext_ significantly depends on three parameters, including *h*′, *η*_int_ ($\eta_{\mathrm{int}}\;=\;\frac{\tau_{l}}{\tau_{r}}$) and *d*_a_, which are used to show PR strongly influences the internal processes in the LEDs. Hence, they demonstrated an increase in *η*_ext_ with a constant active layer thickness (*d*_a_) can be achieved by an increase in *η*_int_ caused by PR. Such an increase is pronounced in LED devices with smaller contact size or halfwidth (*h*′) in which the PR effect is stronger.^[^ ^128^ ^]^

In addition to the theoretical analysis described above, PR, which is capable of improving LED device performance, has already been demonstrated through experimental results.^[^ ^50^, ^129^, ^130^, ^131^, ^132^, ^133^ ^]^ For example, De Neve et al. reported the existence of PR in planar microcavity InGaAs quantum well (QW) LEDs of different sizes.^[^ ^129^ ^]^ They identified why the EQE strongly depends on current density and device size by two main reasons. One is that operating at a low current density can provide narrow intrinsic spectra, which is favorable for the microcavity LEE. Another is that large diameter LEDs guarantee complete reabsorption and recycling of guided modes.^[^ ^129^ ^]^ Figure 9e shows an increase of the EQE due to PR of spontaneous emission (i.e., reabsorption), assuming complete reabsorption of the guided mode, which implies that 30% of the emitted light is reabsorbed by the active layer. If the guided mode is completely reabsorbed and the IQE is close to unity, PR would increase the LEE by 50%. To take advantages of PR, they fabricated a 1.5 mm‐diameter microcavity LED, which yielded an experimental EQE of 19.8% at a current density of 8 A cm^−2^. However, at the same low current density, the EQE of a 85 µm‐diameter LED was only 16.8%, suggesting that PR in LEDs with this diameter was partial.^[^ ^129^ ^]^

Recently, perovskite LEDs have attracted considerable research interests because they have broken the 20% barrier for EQE.^[^ ^134^, ^135^, ^136^ ^]^ However, these high values of EQE cannot be explained by classical models for optical outcoupling. Therefore, Cho et al. verified the contribution and analyzed the role of PR in assisting light extraction from perovskite LEDs. Actually, when the radiation efficiency is sufficiently high, repetitive reabsorption and re‐emission of photons trapped in substrate and waveguide modes significantly enhance light extraction. In state‐of‐the‐art perovskite LEDs with EQE > 20%, they calculated that PR is able to contribute to over 70% of light emission. Finally, they proposed several photonic structures to further improve the performance of perovskite LEDs through maximizing the PR effect.^[^ ^137^ ^]^ Even though the PR of LEDs occurs with unity IQE, the optical energy loss occurring in this process due to the large refractive index of perovskite materials^[^ ^138^ ^]^ must be taken into account when determining the optimum choice of wavelengths for highest efficiency. As discussed above, photons that are trapped within LEDs can be recycled many times and have numerous opportunities to escape. Therefore, PR plays an important role in enhancing the EQE of LEDs.

### 5.3 Photon Recycling in Solar Cells

Studies of PR are most commonly applied to solar cells. According to previous research, PR in solar cells refers to charge carrier generation in the PV active material by reabsorption of photons that originated from radiative recombination.^[^ ^12^, ^60^, ^139^, ^140^ ^]^ Although radiative recombination is inherently present in all PV materials, PR is only relevant to solar cells employing absorber materials with very low nonradiative recombination losses and high internal PLQEs.^[^ ^1^, ^6^, ^12^, ^141^ ^]^ When efficient PR is exploited, solar cells are able to benefit from a strong enhancement in *V*_oc_.^[^ ^65^ ^]^

Typically, the maximum PCE of a solar cell is defined by the SQ limit,^[^ ^142^ ^]^ which relies on the assumptions: a) the probability for the absorption of solar light by the generation of a single electron–hole pair is unity for all photon energies larger than the band gap energy of the PV absorber material; b) the only loss mechanism is spontaneous emission of photons by radiative recombination of electron–hole pairs as required by the principle of detailed balance; (c) the collection probability for all photogenerated electron‐hole pairs is unity.^[^ ^143^, ^144^, ^145^ ^]^ Realistically, PR in solar cells is capable of reducing the loss of photons regenerated from radiative recombination of electron‐hole pairs, and achieving a probability for the absorption of solar light and a collection probability for all photogenerated electron–hole pairs much closer to unity. Hence, PR can play an important role in enhancing the PCE and improving the performance of solar cells.

#### 5.3.1 The Influence of Photon Recycling on the *V*_oc_ of Solar Cells

In recent years, the efficiency of PV devices under 1‐sun illumination, especially single‐junction PV devices, has approached the SQ limit^[^ ^142^, ^146^ ^]^ by managing photon flux within the active layers of solar cells, in part, using PR.^[^ ^65^ ^]^ In fact, the benefit from PR depends on the minimization of the photon flux emitted by the solar cell to its external environment.^[^ ^18^, ^20^, ^64^, ^65^, ^67^ ^]^ The photon flux can be contained in the active layer of a solar cell through limiting the escape cone from the front surface of the device and reducing the photon flux transmitted through the rear of the device.^[^ ^65^ ^]^ Therefore, PR does not affect *J*_sc_ because light emission is quenched at short circuit by efficient charge carrier extraction. However, PR in solar cells can increase *V*_oc_ because it increases the photon path length of the luminescence that effectively reduces the radiative recombination component of the saturation current in a cell.^[^ ^65^, ^144^ ^]^ According to the first study of thermodynamic principles from Ross in 1967^[^ ^147^ ^]^ and its later more extensive derivations and explanations of the equation,^[^ ^67^, ^148^, ^149^ ^]^ *V*_oc_ is correlated with the external radiative efficiency (*ERE*) of a solar cell as follows:

(20) $$V_{\mathrm{oc}}=V_{\mathrm{oc}}^{\mathrm{ideal}}\frac{kT_{c}}{q}\ln\left(\mathrm{ERE}\right)$$

where $V_{\mathrm{oc}}^{\mathrm{ideal}}$ is the detailed balance limited (also known as the SQ limited) *V*_oc_ of an ideal solar cell, *k* is the Boltzmann constant and *T*_c_ is the cell temperature. From Equation (20), it is apparent that a higher *ERE* shifts the solar cell closer to its detailed balance limit for *V*_oc_ (for example, the maximum *ERE* value of 1 would yield *V*_oc_ = $V_{\mathrm{oc}}^{\mathrm{ideal}}$; smaller *ERE* reduces *V*_oc_). Furthermore, *ERE*, defined as the fraction of the total dark current recombination in the device that results in radiative emission from the device,^[^ ^141^ ^]^ can be calculated through solar cell figures of merit and its spectral EQE, as follows:

(21) $$\mathrm{ERE}=\frac{2\pi q}{h^{3}c^{2}}\left(\frac{1}{J_{\mathrm{sc}}}\right)\exp\left(\frac{qV_{\mathrm{oc}}}{kT_{c}}\right)\underset{E_{G}}{\int^{\infty }}\frac{\bar{\mathrm{EQE}}\times E^{2}}{\exp\left(\frac{E}{kT_{c}}\right)-1}\mathrm{dE}$$

In Equation (21), *h* denotes the Planck constant, *c* the speed of light, *E*_G_ the active material band gap energy, *E* is the photon energy, and $\bar{\mathrm{EQE}}$ is the appropriately weighted value of EQE over all angles of incident light. Generally, the ERE is smaller than the internal radiative efficiency (IRE), which is defined as the fraction of the internal recombination events that are radiative, such as is determined by the ratio of total lifetime to the radiative lifetime, due to total internal reflection and PR. Moreover, Equation (21) is closely related to Equation (2) because they both start with the initial research on detailed balance, but one uses energy and another uses frequency.

Since it has been reported that PR can be achieved in metal halide perovskite‐based solar cells and layers,^[^ ^29^ ^]^ Kirchartz et al. studied the impact of PR on the *V*_oc_ of perovskite solar cells and explained how the *V*_oc_ boost from PR depends on the optical and electronic properties.^[^ ^30^ ^]^ For perovskite solar cells, in the absence of parasitic absorption, there are two options for photons created by radiative recombination, namely reabsorption with the probability *p*_r_ and emission with the probability *p*_e_ mentioned in Equation (18). Actually, for PR to be of any relevance, there must be a high probability for light to be reabsorbed, which means that *p*_r_ must be large and *p*_e_ must be small. They derived an equation for the emission probability, *p*_e_, as

(22) $$p_{e}=\frac{\int_{0}^{\infty }a\varphi_{\mathrm{bb}}\mathrm{dE}}{\int_{0}^{\infty }4\alpha ''d'n_{r}^{2}\varphi_{\mathrm{bb}}\mathrm{dE}}$$

where *a* is the absorptance (the fraction of all incident photons that are absorbed), *φ*_bb_ is the blackbody spectrum, *n*_r_ is the refractive index, *α"* is the absorption coefficient of the absorber material and *d'* is the absorber thickness. Using Equation (22), the difference in *V*_oc_, Δ$V_{\mathrm{oc}}^{\mathrm{PR}}$, when taking PR into account relative to the case where it is neglected, can be calculated as follows:

(23) $$\Delta V_{\mathrm{oc}}^{\mathrm{PR}}=-\frac{kT_{c}}{q}\ln\left(p_{e}\right)=\frac{kT_{c}}{q}\ln\left(\frac{\int_{0}^{\infty }4\alpha "d'n_{r}^{2}\varphi_{\mathrm{bb}}\mathrm{dE}}{\int_{0}^{\infty }a\varphi_{\mathrm{bb}}\mathrm{dE}}\right)$$

According to the Equation (23), the change in *V*_oc_ due to PR is negligible if the emission probability is large. Otherwise, there is an increase in *V*_oc_ when emission probability decreases. If the absorptance and the quantum efficiency are identical, the *V*_oc_ in the radiative limit ($V_{\mathrm{oc}}^{\mathrm{rad}}$) can be expressed as

(24) $$V_{\mathrm{oc}}^{\mathrm{rad}}=V_{\mathrm{oc}}^{\mathrm{ideal}}=\frac{kT_{c}}{q}\ln\left(\frac{\int_{0}^{\infty }a\varphi_{\mathrm{sun}}\mathrm{dE}}{\int_{0}^{\infty }a\varphi_{\mathrm{bb}}\mathrm{dE}}\right)$$

where *φ*_sun_ is the solar spectrum at room temperature. Then, Kirchartz et al. found that closer to the radiative limit, parasitic absorption becomes a major factor to reduce the possible *V_oc_*. Thus, they further derived an analytical equation on the basis of Equation (20):

(25) $$\begin{array}{ccc} qV_{\mathrm{oc}} & = & qV_{\mathrm{oc}}^{\mathrm{rad}}+kT_{c}\ln\left\{Q_{e}^{\mathrm{lum}}\right\}\\ & = & qV_{\mathrm{oc}}^{\mathrm{rad}}+kT_{c}\ln\;\left\{\frac{p_{e}Q_{i}^{\mathrm{lum}}}{\left(1-Q_{i}^{\mathrm{lum}}\right)+\left(p_{e}+p_{a}\right)Q_{i}^{\mathrm{lum}}}\right\}\\ & = & qV_{\mathrm{oc}}^{\mathrm{rad}}+kT_{c}\ln\left\{\frac{p_{e}Q_{i}^{\mathrm{lum}}}{1-p_{r}Q_{i}^{\mathrm{lum}}}\right\} \end{array}$$

that sums up the effects of reabsorption, emission and parasitic absorption on the *V*_oc_ of a solar cell in the presence of PR. Here, $V_{\mathrm{oc}}^{\mathrm{rad}}$ is the radiative *V*_oc_ assuming no concentration or restriction of light emission and $Q_{e}^{\mathrm{lum}}$ and $Q_{i}^{\mathrm{lum}}$ are the external and internal luminescence quantum efficiencies, respectively. It should be noted here that Equation (25) is more accurate than Equation (20) to indicate how to influence *V*_oc_ in devices because the possibilities for emission, parasitic absorption and reabsorption are considered together. However, ERE is closely related to the external quantum efficiency of a light‐emitting diode operating at a similar applied voltage but not the same. Thus, the external luminescence quantum efficiency$\;(Q_{e}^{\mathrm{lum}}$) mentioned in Equation (25) is somewhat inappropriate and controversial. The probabilities for emission and parasitic absorption are *p*_e_ and *p*_a_, respectively. Together with the probability *p*_r_ for reabsorption, the sum of these three probabilities should be 1. Finally, they defined the voltage loss Δ$V_{\mathrm{oc}}^{\mathrm{pa}}$ due to parasitic absorption

(26) $$\Delta V_{\mathrm{oc}}^{\mathrm{pa}}=-\frac{kT_{c}}{q}\ln\left\{\frac{p_{e}}{p_{e}+p_{a}}\right\}$$

Furthermore, according to the previous approximations by a non‐rigorous treatment of *p*_a_ and *p*_e_ from Kirchartz et al., Abebe et al. derived an explicit expression for Δ$V_{\mathrm{oc}}^{\mathrm{PR}}$ with rigorous wave‐optical treatment, which revealed its dependence on $Q_{i}^{\mathrm{lum}}$, parasitic reabsorption, and the escape probability of radiatively emitted photons.^[^ ^12^ ^]^ They relied on rigorous calculation of Δ$V_{\mathrm{oc}}^{\mathrm{PR}}$ in arbitrary single‐junction solar cells in the presence of nonradiative recombination and parasitic photon reabsorption. The equation is expressed as follows:

(27) $$q\Delta V_{\mathrm{oc}}^{\mathrm{PR}}=kT_{c}\ln\left\{\frac{1}{1-\left(1-p_{e}-p_{a}\right)Q_{i}^{\mathrm{lum}}}\right\}$$

As a result, the boost of *V*_oc_ due to PR should be relevant if recombination at the surfaces and parasitic absorption are sufficiently small. This boost would be reduced if light trapping was implemented because light trapping also improves light outcoupling and thereby reduces the percentage of reabsorbed photons that could further increase the carrier concentration and thereby the photovoltage.^[^ ^30^ ^]^ Increased relevance of PR will imply that types of nonradiative recombination become important that would not be relevant far from the radiative limit.^[^ ^30^ ^]^ In particular, parasitic absorption of photons emitted by the solar cell absorber in contact layers will be important to minimize in order to fully benefit from voltage enhancements arising from PR.^[^ ^12^ ^]^

#### 5.3.2 Photon Recycling in Different Types of Solar Cells

To date, PR has been applied in crystalline silicon solar cells,^[^ ^13^, ^14^, ^15^, ^16^, ^68^, ^147^, ^148^, ^149^, ^150^, ^151^, ^152^, ^153^ ^]^ III‐V materials solar cells,^[^ ^31^, ^37^, ^38^, ^45^, ^59^, ^64^, ^66^, ^71^, ^92^, ^93^, ^140^, ^154^, ^155^, ^156^, ^157^, ^158^, ^159^, ^160^, ^161^, ^162^, ^163^, ^164^, ^165^, ^166^, ^167^, ^168^, ^169^, ^170^, ^171^, ^172^ ^]^ organic solar cells^[^ ^175^, ^176^ ^]^ and perovskite solar cells.^[^ ^28^, ^29^, ^87^, ^95^, ^111^, ^177^, ^178^, ^179^, ^180^, ^181^, ^182^, ^183^, ^184^, ^185^, ^186^, ^187^, ^188^, ^189^, ^190^, ^191^, ^192^, ^193^, ^194^ ^]^ In this section, we review the calculations and applications of PR in different types of solar cells. **Table** **3** summarizes different kinds of solar cells that exhibit PR and their main characteristics. It has been reported that no matter what types of solar cells, single‐junction or multi‐junction solar cells, PR is capable of increasing the value of *V*_oc_ to enhance the total PCE.

**Table 3:** Summary of different solar cells in which PR is demonstrated

Active materials	T/E	Thickness of active layer	*V*_oc_ [mV]	*J*_sc_ [mA cm^−2^]	FF [%]	PCE [%]	Reference No.
Si	T	90 µm	720	‐	‐	29.05	^[^ ^14^ ^]^
Si	T	110 µm	761.3	43.31	89.26	29.43	^[^ ^16^ ^]^
Si	T	100 µm	769	52.2	89	29.8	^[^ ^147^ ^]^
Si	T	1 µm	720	‐	‐	19.8	^[^ ^148^ ^]^
Si	T	400 µm	720	‐	‐	26.2	^[^ ^148^ ^]^
Si	T	98.1 µm	763.3	43.36	89.31	29.56	^[^ ^150^ ^]^
Si	E	165 µm	744	42.3	83.8	26.3	^[^ ^68^ ^]^
AlGaAs/GaAs	T	1.6 µm	1286	‐	89.69	36.41	^[^ ^38^ ^]^
InP	T	5 µm	1091	42.1	89	30.2	^[^ ^45^ ^]^
AlGaAs/GaAs	T	1.65 µm	1107	29.49	86.7	28.35	^[^ ^64^ ^]^
GaAs	E	2.975 µm	1075	29.57	79.95	25.41	^[^ ^65^ ^]^
GaAs	E	2.975 µm	1069	29.65	80.47	25.51	^[^ ^65^ ^]^
GaAs	T	2.65 µm	1230	‐	85.5	28.8	^[^ ^66^ ^]^
GaAs	T	‐	1395	‐	‐	‐	^[^ ^71^ ^]^
GaAs	E	‐	1124	‐	‐	‐	^[^ ^71^ ^]^
GaAs	T	2 µm	1101	29.46	85.76	27.81	^[^ ^92^ ^]^
GaAs	T	3.5 µm	1069	29.9	82.4	26.3	^[^ ^93^ ^]^
GaAs	E	205 nm	1022	24.64	79.2	19.9	^[^ ^94^ ^]^
InP	T	280 nm	1120	28.2	88.3	27.9	^[^ ^160^ ^]^
GaAs	E	‐	1083	‐	‐	‐	^[^ ^151^ ^]^
GaInP	E	1 µm	1452	15.8	89.4	20.5	^[^ ^164^ ^]^
GaInP/GaAs	E	‐	2500	13.87	‐	30.21	^[^ ^169^ ^]^
GaInAsP/GaInAs	E	‐	1052	11.16	‐	8.54	^[^ ^169^ ^]^
Perovskite	E	350 nm	1060	22	69	16.3	^[^ ^87^ ^]^
Perovskite	T	200 nm	1325	22.27	91	26.77	^[^ ^95^ ^]^
Perovskite	T	500 nm	1315	25.27	91	30.06	^[^ ^95^ ^]^
Perovskite	T	1000 nm	1305	25.97	91	30.69	^[^ ^95^ ^]^
Perovskite	T	200 nm	1300	25.38	91	29.86	^[^ ^95^ ^]^
Perovskite	T	500 nm	1295	26.13	91	30.59	^[^ ^95^ ^]^
Perovskite	T	1000 nm	1290	26.46	91	30.88	^[^ ^95^ ^]^
Perovskite	T&E	80 nm	890	14.86	75	9.92	^[^ ^195^ ^]^
Perovskite	T&E	240 nm	900	22.11	80	15.92	^[^ ^195^ ^]^
Perovskite	T&E	200 nm	910	22.97	80	16.72	^[^ ^195^ ^]^
Perovskite	T&E	200 nm	880	18.63	69	11.31	^[^ ^195^ ^]^
Perovskite	T&E	200 nm	940	20.14	66	12.49	^[^ ^195^ ^]^

**Note**: T = Theoretical; E = Experimental.

##### Photon Recycling in Crystalline Silicon Solar Cells

**Figure 11:**
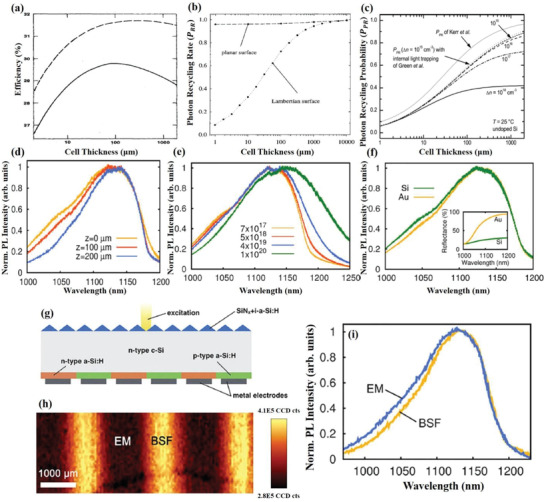
a) Efficiency of crystalline silicon solar cells as a function of thickness for textured cells with back reflectors. The top curve includes only radiative recombination and the bottom curve includes Auger recombination and free carrier absorption in addition to radiative recombination. b) Calculated values for the PR rate *P*_RR_ in crystalline silicon as a function of cell thickness for Lambertian surface and planar surface. c) PR probability (*P*_PR_) for undoped silicon as a function of the wafer thickness for different injection levels Δ*n*, also including *P*_RR_ calculated assuming the Lambertian light trapping according to Tiedje et al.,^[^ ^147^ ^]^ Green et al.^[^ ^153^ ^]^ and Kerr et al.^[^ ^14^ ^]^ d) Normalized confocal micro‐PL spectra recorded from a commercial p‐type crystalline silicon wafer at different depth distances from the front surface. e) Normalized PL spectra recorded from p‐type crystalline silicon wafers with different doping levels. f) Normalized averaged PL spectra corresponding to the region inside the Au deposited layer and outside the Au region (crystalline silicon wafer). The inset shows the reflectance of both regions for the spectral range. g) Scheme of the measured IBC solar cell. h) Reconstructed PL intensity map of the emitter and BSF regions. i) Normalized averaged PL spectra corresponding to the emitter and BSF regions. a) Reproduced with permission.^[^ ^147^ ^]^ Copyright 1984, IEEE. b) Reproduced with permission.^[^ ^14^ ^]^ Copyright 2003, Wiley‐VCH. c) Reproduced with permission.^[^ ^16^ ^]^ Copyright 2013, IEEE. d–i) Reproduced with permission.^[^ ^151^ ^]^ Copyright 2017, AIP Publishing.

There is no doubt that silicon is uniquely favorable as a PV material because it is one of the most abundant elements in nature and it is an elemental semiconductor with an absorption spectrum that overlaps with most of the solar spectrum.^[^ ^147^ ^]^ Tiedje et al. calculated the limiting efficiency of crystalline silicon solar cells as a function of silicon thickness, based on their measured optical absorption of crystalline silicon, for hypothetical solar cells in which the fundamental loss mechanism, radiative recombination, Auger recombination, and free carrier absorption are operative.^[^ ^147^ ^]^ They assumed the surfaces of the silicon are textured to fully randomize the incident sunlight for maximum enhancement of the optical absorption through light trapping and PR of recombination radiation.^[^ ^147^ ^]^ **Figure** **11**a showed the efficiencies of crystalline silicon solar cells with different thickness. As a result, the maximum limiting efficiency was calculated to be 29.8% for a crystalline silicon solar cell with thickness around 100 nm. In fact, it is approaching the maximum value that can be obtained because the limiting efficiency for an ideal radiative‐recombination‐limited silicon solar cell is 32.9%.^[^ ^147^ ^]^ Considering the Coulomb‐enhanced (CE) Auger recombination for highly injected and high‐resistivity silicon, Green updated the limiting efficiency to 28.8% for an 80 µm‐thick solar cell and an efficiency of 24.7% is feasible even for silicon solar cells with a thickness of 1 µm with an isotropic response and a Lambertian reflector.^[^ ^14^, ^15^, ^148^ ^]^

Furthermore, Kerr et al. applied a new parameterization incorporating CE Auger recombination, which accurately fits the experimental Auger lifetime data for arbitrary injection levels, to investigate the effect of cell thickness on modelling the limiting efficiency of crystalline silicon solar cells, including the effects of PR.^[^ ^13^, ^14^, ^15^, ^149^ ^]^ Based on their Auger parameterization, the *J*–*V* characteristic for a crystalline silicon solar cell limited by CE intrinsic recombination processes is:

(28) $$\begin{array}{ccc} & & J\;=J_{L}-qWn_{i}^{2}\exp\left(\frac{qV}{kT}\right)\\ & & \left\{\;1.8\;\times 10^{-24}n_{0}^{0.65}+6\;\times 10^{-25}p_{0}^{0.65}+3\;\times 10^{-27}\times {\left\{0.5\left[\sqrt{{\left(n_{0}+p_{0}\right)}^{2}+4n_{i}^{2}\exp\left(\frac{qV}{kT}\right)}-\left(n_{0}+p_{0}\right)\right]\right\}}^{0.8}+\;\left(1-P_{\mathrm{RR}}\right)\;\times \;9.5\;\times 10^{-15}\right\} \end{array}$$

where *n_i_* is the intrinsic carrier concentration ( *n_i_* = *n*_0_  + Δ*n* = *p*_0_ + Δ*n*), *n*_0_ and *p*_0_ are the equilibrium concentrations of electrons and holes and Δ*n* is the density of excess electrons, which is equal to the density of excess holes.^[^ ^14^, ^15^, ^149^ ^]^ *J*_L_ is the light‐generated current density, which was determined assuming a Lambertian light trapping scheme^[^ ^147^, ^148^, ^150^ ^]^ using the AM 1.5G spectrum at 25 °C normalized to an illumination intensity of 0.1 W cm^−2^ and *W* is the cell thickness. Note that Δ*n* and *W* are also mentioned in Equations (5) and (3), respectively. In Equation (28), *P*_RR_ gives the PR rate, which accounts for the fact that radiative recombination results in the emission of photons that can be reabsorbed or recycled in the crystalline silicon. The current density *J* will increase with an increased *P*_RR_. Figure 11b shows that *P*_RR_ is dependent on the cell thickness with a Lambertian reflector, but it is virtually independent for planar surfaces.^[^ ^14^ ^]^ According to Figure 11c, Kerr et al. demonstrated that the losses from radiative recombination are negligible for a thick cell because most of the emitted photons are recycled, while the losses from radiative recombination are significant for a thin cell due to the low *P*_RR_.^[^ ^14^, ^15^ ^]^ On the other hand, for planar surfaces, *P*_RR_ is high and independent of cell thickness because the emitted photons are trapped by total internal reflection at the silicon‐air interface and remain trapped until they are reabsorbed.^[^ ^14^ ^]^ As a result, the maximum achievable efficiency calculated by this new parameterization is 29.05% for a 90 µm‐thick solar cell made from high‐resistivity crystalline silicon at 25 °C.

In 2013, Richter et al. also calculated the limiting efficiency for crystalline silicon solar cells under the AM 1.5G at 25 °C, but they addressed the effect on the efficiency limit for each parameter separately, which differs from the calculations of Kerr.^[^ ^16^ ^]^ They determined PR probability (*P*_PR_) by evaluating numerically

(29) $$P_{\mathrm{PR}}\left(W,\;n,\;p\right)=\frac{\int_{0}^{\infty }A_{\mathrm{bb}}\left(E,\;W,\;n,\;p\right)B\left(E\right)dE}{\int_{0}^{\infty }B\left(E\right)dE}$$

where *n* and *p* are mentioned in Equation (3), *B*(*E*) is the spectrally resolved radiative recombination coefficient calculated from the generalized Planck equation with the photon energy *E*. *A*_bb_(*E*, *W*,  *n*,  *p*) is the spectrally resolved relative absorbance as a function of *E*,  *W*,  *n*,  and *p*.^[^ ^16^ ^]^ Figure 11c shows *P*_PR_ for undoped silicon as a function of the cell thickness *W* for different injection levels Δ*n*. It is obvious that *P*_PR_ decreased when Δ*n* increased because of the increasing parasitic free carrier absorption (FCA). Based on the new calculations, they presented a maximum theoretical efficiency of 29.43% for a 110 µm‐thick undoped crystalline silicon solar cell.^[^ ^16^ ^]^ Similarly, Schäfer and Brendel further calculated the limiting efficiency for crystalline solar cells based on the commonly applied weak absorption approximation from Richter et al. of Lambertian light trapping.^[^ ^150^ ^]^ The maximum efficiency from their calculations was enhanced to 29.56% and the optimum wafer thickness reduced to 98.1 µm for undoped crystalline silicon while using the same material parameters and the same solar spectrum as Richter et al.^[^ ^150^ ^]^

Experimentally, Roigé et al. presented a comprehensive study about the effects and implications of PR on confocal micro‐PL measurements in crystalline silicon to analyze how the PR phenomenon is affected by different configurations and to quantify how the process affects the emission line‐shape of the resulting micro‐PL spectra.^[^ ^151^ ^]^ First, they recorded the normalized confocal micro‐PL spectra in backscatter mode from a p‐type crystalline silicon wafer by changing the distance, *z*, between objective lens and the sample surface (Figure 11d). As it can be seen, the level of PR increases with increasing the depth distance from 0 (on focus) to 200 µm, which means that less short wavelength emission is attributed to stronger reabsorption at shorter wavelengths. Furthermore, they recorded the normalized PL spectra at room temperature from p‐type crystalline silicon wafers with different doping densities (Figure 11e). They found that the PL emission spectrum starts to widen progressively towards long wavelengths for high doping densities because of band gap narrowing, which is attributed to band gap renormalization. In addition, another observation is the small but progressive decrease of the PL intensity between 1000 and 1050 nm with decreasing doping densities.^[^ ^152^ ^]^ Since the fact that increasing doping densities results in the increase of the silicon absorption coefficient,^[^ ^154^ ^]^ doping densities of crystalline silicon wafers affect PR.^[^ ^151^ ^]^

In addition, Roigé et al. sputtered a thin Au layer onto the back surface of a SiO_2_‐passivated p‐type crystalline silicon wafer to study the impact of surface reflectance on PR. Figure 11f shows the normalized and averaged PL spectra corresponding to both regions, inside the Au deposited area and outside the Au region (crystalline Si wafer). The inset in Figure 11f is the reflectance of both regions. By comparing the PL intensity in the spectral range of 1000–1100 nm, they determined that high values of reflectance lead to an increase of recycling.^[^ ^151^ ^]^ Finally, they demonstrated that the surface recombination velocity (SRV) can also have implications in the recycling level and illustrated the effect of SRV on recycling with an interdigitated back contacted (IBC) silicon solar cell, schematically shown in Figure 11g. For an IBC solar cell, an a‐Si:H(i)/a‐Si:H(n) stack and an a‐Si:H(i)/a‐Si:H(p) stack, which were deposited by plasma enhanced chemical vapor deposition at 200 °C, were used to form the back‐surface field (BSF) and the emitter, respectively. Figure 11h shows the reconstructed PL intensity map of the selected IBC solar cell area, where two regions, emitter and BSF, are clearly identified. They found that BSF regions are associated with a lower SRV than emitter regions due to a higher PL intensity in Figure 11h. Therefore, according to the variations in the 1000–1100 nm wavelength range shown in Figure 11i, the lower SRV associated with the BSF regions leads to a subsequent increase of the radiative recombination, which enhances the recycling of photons coming from the back surface.^[^ ^151^ ^]^

##### Photon Recycling in III–V Materials Solar Cells

Early research into multijunction PVs leveraged the properties of semiconductors comprised from elements in the III and V columns of the Periodic Table, especially gallium indium phosphate (GaInP), gallium indium arsenide (GaInAs), gallium arsenide (GaAs), and indium phosphate (InP) because the maximum efficiencies of III–V multijunction devices have reached over 40%.^[^ ^31^, ^37^, ^38^, ^45^, ^66^, ^92^, ^93^, ^140^, ^154^, ^155^, ^156^, ^157^ ^]^ Nowadays, PR plays an important role in improving the limit efficiencies of III–V materials solar cells.^[^ ^38^, ^59^, ^64^, ^71^, ^158^, ^159^, ^160^, ^161^, ^162^, ^163^, ^164^, ^165^, ^166^, ^167^, ^168^, ^169^, ^170^, ^171^, ^172^ ^]^

**Figure 12:**
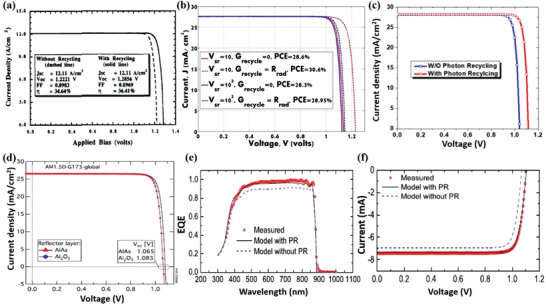
a) Illuminated (500 Suns, AM 1.5 direct) *J*–*V* characteristics of thin‐film GaAs solar cell for two separate cases: neglecting PR and including PR. b) *J*–*V* curves of the GaAs solar cells with and without PR. Blue curves and red curves correspond to the case when *V*_sr_= 10 cm s^−1^ and *V*_sr_= 10^3^ cm s^−1^, respectively. c) Simulated *J*–*V* curve of the proposed heterojunction solar cell with and without PR. d) Measured 1‐sun *J*–*V* curve of the GaAs solar cells with reflector before (AlAs) and after (Al_2_O_3_) oxidation, which means that the effect of PR exists or not. Measured and modeled values of e) EQE and f) *I*–*V* characteristics under the AM 1.5G reference spectrum for a GaAs solar cell with and without PR. a) Reproduced with permission.^[^ ^38^ ^]^ Copyright 1994, IEEE Publishing Group. b) Reproduced with permission.^[^ ^159^ ^]^ Copyright 2016, Nature Publishing Group. c) Reproduced with permission.^[^ ^160^ ^]^ Copyright 2020, IEEE. d) Reproduced with permission.^[^ ^161^ ^]^ Copyright 2014, AIP Publishing. e,f) Reproduced with permission.^[^ ^163^ ^]^ Copyright 2014, AIP Publishing.

At the beginning, a lot of research studied the effect of PR in III–V materials solar cells through theoretical calculations. In 1994, Durbin and Gray showed the *J* *‐V* characteristics of the thin GaAs solar cells under illumination (500 Suns, AM 1.5 direct) both with and without PR in **Figure** **12**a. It is apparent that the performance of devices including PR was much better than those neglecting PR.^[^ ^38^ ^]^ Furthermore, Eyderman and John studied the effect of PR on PCEs of GaAs photonic crystal solar cells by numerical solutions of the coupled electromagnetic Maxwell and semiconductor drift‐diffusion equations.^[^ ^159^ ^]^ The results are shown in Figure 12b, which are the *J*–*V* characteristics for a surface recombination velocity (*V*_sr_) of 10^3^ cm s^−1^ and 10 cm s^−1^. If all light generated by radiative recombination is assumed to escape the device, the PCE is 28.3% for *V*_sr_ = 10^3^ cm s^−1^, whereas it is 28.6% for *V*_sr_ = 10 cm s^−1^. On the other hand, when all radiative recombination is recycled (*R*_rad_ = *G*_recycle_), for different *V*_sr_ of 10^3^ cm s^−1^ and 10 cm s^−1^, the PCEs of these two cases increases to 29% and 30.6%, respectively.^[^ ^159^ ^]^ In addition, it is also confirmed that the enhanced role of PR with the suppression of nonradiative decay provides a significant increase in *V*_oc_. The *V*_oc_ is obtained by the expression:

(30) $$V_{\mathrm{oc}}=\frac{kT}{q}\ln\left(\frac{I_{\mathrm{sc}}}{I_{\mathrm{sat}}}+1\right)$$

In Equation (30), *I*_sc_ is the short‐circuit photocurrent (when *V* = 0) and *I*_sat_ is a saturation current that is determined by carrier recombination. When *V*_sr_ = 10 cm s^−1^, Equation (30) yields *V*_oc1_ = 1.18 V with no PR and *V*_oc2_ = 1.29 V with perfect PR. On the other hand, when *V*_sr_ = 10^3^ cm s^−1^, Equation (30) yields *V*_oc3_ = 1.15 V with no PR and *V*_oc4_ = 1.17 V with perfect PR.^[^ ^159^ ^]^ Recently, a 1D device simulation performed on a InP heterojunction solar cell shows that PCE of 26.1% and 27.6% can be achieved with and without PR, respectively, under ideal conditions.^[^ ^160^ ^]^ Figure 12c shows the *J*–*V* curves with and without PR. As expected, PR improved the *V*_oc_ from 1.05 to 1.12 V, while maintaining the *J*_sc_ and fill factor (FF) at 28.2 mA cm^−2^ and 88.3%, respectively.^[^ ^160^ ^]^

As for how to achieve PR in III–V materials solar cells, for example, Gruginskie et al. reported that the performance of thin‐film GaAs solar cells was increased by PR using a rear mirror.^[^ ^59^ ^]^ Particularly, the total reflectance at the back of the solar cell in this case was increased from 63.5% to 93.2%. In addition, García et al. reported that they used metal or low‐index dielectric‐based back reflectors to confine the re‐emitted photons and enhance PR.^[^ ^161^ ^]^ In this case, Al_2_O_3_‐based reflectors, created by lateral oxidation of an AlAs layer, are identified as a feasible choice for on‐substrate solar cells, which can produce a *V*_oc_ increase of around 65% of the maximum increase attainable with an ideal reflector. In Figure 12d, it is obvious that a *V*_oc_ increase of a GaAs solar cell with an Al_2_O_3_‐based reflector is about 18 mV (≈2%) more than a solar cell with an AlAs‐based reflector because of the existence of PR.^[^ ^161^ ^]^ Furthermore, Lumb et al. outlined an approach to account for PR in the analytical Hovel model^[^ ^162^ ^]^ and compared the analytical model to experimental data for high‐quality GaAs‐based solar cells in order to demonstrate that PR is a necessary component to reproduce the performance of solar cells operating close to the fundamental limit.^[^ ^163^ ^]^ For comparison, the modeled value of EQE and *I*–*V* characteristics with and without PR are shown in Figure 12e,f, respectively. When PR is neglected, the EQE and *V*_oc_ values are lower because the diffusion length for holes in the emitter is reduced, which reduces collection efficiency.^[^ ^163^ ^]^

##### Photon Recycling in Organic Solar Cells

**Figure 13:**
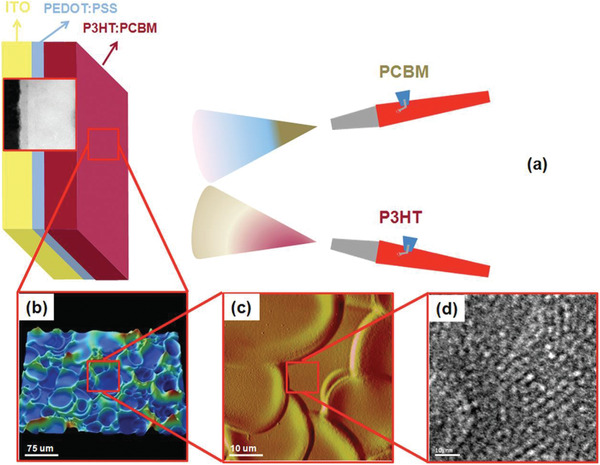
a) Schematic of the alternating spray deposition apparatus. The device structure is shown with a TEM cross‐sectional image, indicating the ITO/PEDOT:PSS/P3HT:PCBM layers starting from the left with well‐defined interfaces. Representative images taken from b) optical profiler, c) AFM, and d) TEM of 150 °C annealed film from P3HT/PCBM alternating deposition. a–d) Reproduced with permission.^[^ ^175^ ^]^ Copyright 2010, American Chemical Society.

**Figure 14:**
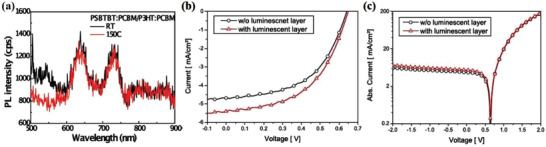
a) PL spectra for the three‐component heterojunction films spray‐coasted from alternating P3HT:PCBM/PSBTBT:PCBM solutions before and after annealing at 150 °C. b,c) are linear and logarithm *J*–*V* curves of the OSCs with and without a luminescent layer, respectively. a) Reproduced with permission.^[^ ^175^ ^]^ Copyright 2010, American Chemical Society. b,c) Reproduced with permission.^[^ ^176^ ^]^ Copyright 2012, AIP Publishing.

Since 1990s, there has been great interest in the development of low‐cost organic solar cells (OSCs) for renewable energy applications.^[^ ^173^, ^174^, ^175^, ^176^ ^]^ Their room‐temperature, solution processibility, and compatibility with flexible, large‐area substrates make OSCs attractive alternatives to many of their inorganic counterparts.^[^ ^173^, ^174^, ^175^, ^176^ ^]^ In fact, PR has not been reported extensively for OSCs. However, there are a few studies that mention the presence of PR effects in the study of OSC morphology and devices. Chen et al. developed an alternating spray deposition method to apply a wide spectrum of organic material systems to substrates, which enables the formation of multi‐component films from independent sources regardless of the distinct material properties. The multi‐component films formed by two organic donor materials can achieve PR in the active layers so that the photoresponse range can complement the solar spectrum.^[^ ^175^ ^]^ **Figure** **13**a showed the alternating spray deposition apparatus, with poly(3‐hexylthiophene) (P3HT) and [6,6]‐phenyl C_61_ butyric acid methyl ester (PCBM) solutions as two material sources. Figure 13b–d showed a series of images obtained from an optical profiler, atomic force microscope (AFM), and transmission electron microscope (TEM), respectively, of films prepared from the alternating spray deposition method after annealing at 150 °C. Since continuous deposition partially dissolved the previous droplets and replenished the liquid solution for resolidfication at the pinned contact line, ring‐like deposits were formed. In Figure 13d, the bright and dark regions resemble the PCBM‐ and P3HT‐rich domains, respectively, by defocusing the TEM to 5 µm. From Figure 13, they explained that it was possible to achieve PR from the nonuniform morphologies. Additionally, they explained that the morphology obtained through this alternating spray deposition holds tremendous potential in realizing PR by two polymer blend systems: P3HT and poly[(4,42‐bis(2‐ethylhexyl)dithieno[3,2‐*b*:22,32‐*d*]silole)‐2,6‐diyl‐*alt*‐(2,1,3‐benzothiadiazole)‐4,7‐diyl] (PSBTBT) blended with PCBM because excess excitons generated in P3HT domains may be reabsorbed with the presence of the lower band gap PSBTBT in the vicinity. In the P3HT:PCBM and PSBTBT:PCBM (5:1) alternating structure, **Figure** **14**a showed that the PL emission at the wavelength of 500–600 nm from P3HT is either quenched or reabsorbed by PSBTBT.^[^ ^175^ ^]^

In the previous research about organic semiconductors, the interaction of UV‐light is one of the major driving factors for degradation. Particularly, photoinduced oxidation severely limits the lifetime in OSCs.^[^ ^176^ ^]^ Therefore, down‐conversion of a part of the blocked UV‐light not only cuts out the UV‐part of the electromagnetic spectrum to seal the devices but also enables recycling of the photons. Engmann et al. presented down‐conversion via a luminescent layer to achieve PR in OSCs across a UV‐blocking TiO_2_ layer.^[^ ^176^ ^]^ Figure 14b,c showed the *J*–*V* curves of the best devices they fabricated with and without a luminescent layer. It is obvious that both *J*_sc_ and *V*_oc_ of devices with a luminescent layer increased to improve the performance because of the strong absorbance of the TiO_2_‐SiO_x_ layer in the UV–vis region, the use of luminescent layer as an anti‐reflection coating and the reabsorption of photons re‐emitted by luminescent layer.^[^ ^176^ ^]^ More evidence is needed to confirm the role of PR in this research because it has been reported that PR can significantly affect the values of *V*_oc_ but not *J*_sc_. Therefore, the conflation of down‐conversion by reabsorption with useful PR in this case is an aspect that is debatable. Compared to studies of PR for conventional inorganic PV devices, there are only a few cases of PR in OSCs; so, the role of PR in OSC is still not clear.

##### Photon Recycling in Perovskite Solar Cells

A class of man‐made materials, called perovskites, have a unique crystallographic structure that makes them highly effective at converting photons of light from the sun into usable electricity. PR has already been invoked to affect various aspects of luminescence‐based experiments performed on lead‐halide perovskite single crystals,^[^ ^177^ ^]^ which have been used as *γ*‐ray and *x*‐ray detectors,^[^ ^178^, ^179^, ^180^ ^]^ photodetectors,^[^ ^181^ ^]^ and gas sensors.^[^ ^182^ ^]^ Previous reports have explained the interpretation of bimolecular recombination in PL transients of the thin films,^[^ ^183^, ^184^ ^]^ the lateral decay of luminescence away from an excitation spot in the thin films and the redshift of transient PL spectra with time.^[^ ^29^, ^185^, ^186^ ^]^

Perovskite solar cells have advanced rapidly, achieving high device efficiencies with significant opportunities to realize a low‐cost, industry‐scalable technology. Although perovskite solar cells have been in development for only a few short years, perovskite solar cells achieved with high PCEs exceeding 20%^[^ ^187^ ^]^ due to merits of the direct band gap,^[^ ^187^, ^188^, ^189^ ^]^ high and balanced mobility,^[^ ^190^ ^]^ long electron‐hole diffusion length,^[^ ^191^, ^192^, ^193^ ^]^ and low nonradiative Auger recombination.^[^ ^194^ ^]^ In order to maximize the efficiencies of perovskite solar cells, one of the most popular methods is to achieve PR.^[^ ^28^, ^29^, ^87^, ^95^, ^111^, ^186^, ^195^, ^196^, ^197^, ^198^, ^199^, ^200^, ^201^, ^202^ ^]^ This is attributed to increased diffusion lengths and concentration of charge carriers and, thereby, increased *V*_oc_ of a PV device.^[^ ^30^, ^120^ ^]^

**Figure 15:**
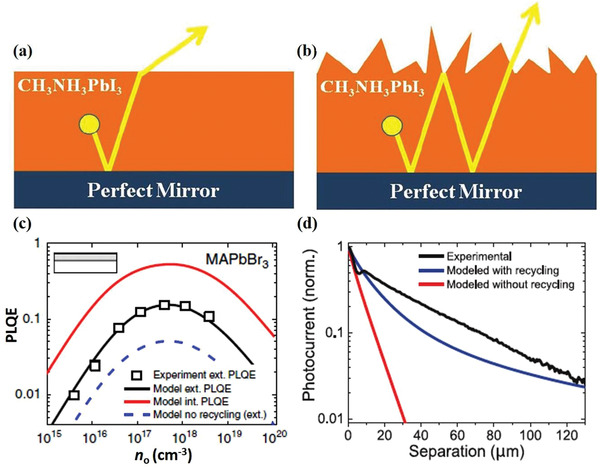
CH_3_NH_3_PbI_3_ perovskite solar cells with different structures: a) a planar front surface with a perfectly reflecting mirror on the rear surface; b) a randomly textured front surface and a perfectly reflecting mirror on the rear surface. c) External PLQEs for MAPbBr3 under pulsed laser excitation (squares) together with the modelled internal (red) and external (black) PLQEs. The internal PLQEs reach values up to 70%. The blue line represents the model without PR. d) Predicted spatial photocurrent decay for the model with and without PR. a,b) Reproduced with permission.^[^ ^95^ ^]^ Copyright 2015, AIP Publishing. c) Reproduced with permission.^[^ ^111^ ^]^ Copyright 2016, Nature Publishing Group. d) Reproduced with permission.^[^ ^29^ ^]^ Copyright 2016, American Association for the Advancement of Science.

Theoretically, Sha et al. predicted the efficiency limit of methylammonium lead iodide (CH_3_NH_3_PbI_3_) perovskite solar cells with the consideration of the effect of PR through a detailed balance model. They calculated both absorptivity for perovskite solar cells, having a planar front surface with a perfectly reflecting mirror on the rear (**Figure** **15**a) and having a randomly textured front surface with a perfectly reflecting mirror on the rear (Figure 15b), to explain how PR influences the device performance. In this case, it is found that the perovskite solar cell having a randomly textured front surface with a perfectly reflecting mirror on the rear has a higher PCE because of the effect of PR, especially if the thickness of the perovskite solar cell is smaller than 400 nm.^[^ ^95^ ^]^ Similarly, Richter et al. reported that PR effects have to be taken into account to accurately determine intrinsic recombination constants and lead to a significant difference between internally and externally measured PLQEs in thin film samples.^[^ ^111^ ^]^ For example, Figure 15c shows both the experimental and modelled external PLQE for bromide perovskite (MAPbBr_3_). Actually, PR is happening in these perovskites because of the modelled internal (red) and external (black) PLQEs, which means PR enables enhanced out‐coupling compared to the external PLQEs (blue) modelled assuming no PR exists.^[^ ^111^ ^]^ Additionally, Pazos–Outón et al. reported PR in lead iodide perovskite solar cells through model analysis and experimental results. According to **Figure** 15d, in order to match the experimental photocurrent, PR has to be taken into account in theoretical models to explain the observed long spatial decays, which means that the average travel distance could be enhanced at larger charge‐carrier densities and can reach values beyond 50 µm.^[^ ^29^ ^]^

Furthermore, Fang et al. quantitatively evaluated the reabsorption and re‐emission processes to determine PR efficiency in hybrid perovskite with its single crystals by measuring the ratio of the re‐emitted photons to the initially excited photons. They reported that the PR efficiencies are revealed to be less than 0.5% in CH_3_NH_3_PbI_3_ and CH_3_NH_3_PbBr_3_ single crystals under excitation intensity close to one sun, highlighting the intrinsically long carrier recombination lifetime instead of the photon‐recycling‐induced photon propagation as the origin of their long carrier diffusion length.^[^ ^28^ ^]^ Recently, Sha et al. built a modified detailed balance model to capture the light‐absorption‐dependent *J*_sc_, contact and transport‐layer‐modified carrier transport, as well as recombination and PR influenced *V*_oc_, which is much simpler than the drift‐diffusion model and considers the PR effect.^[^ ^95^, ^195^ ^]^ In order to study the loss mechanism and quantify loss factors, the photocurrent (*I'*) in the revised detailed balance model is expressed as

(31) $$I'\;=\;\frac{V-I'R_{s}}{R_{\mathrm{sh}}}+I_{n}\left(V-I'R_{s}\right)+I_{r}\left(V-I'R_{s}\right)-I_{p}$$

where *V* is the applied voltage of a solar cell, *I*_p_ is the photocurrent, *I*_r_ and *I*_n_ are the current loss due to radiative emission by PR and the nonradiative recombination by defects and impurities, respectively. *R*_s_ is the series resistance to describe the ohmic loss. The ohmic loss is cause by the contacts, carrier transport layers and the heterojunction interfaces between the perovskite and carrier transport layers. *R*_sh_ is the shunt resistance, which represents the defects and void‐induced current leakage. In contrast to the drift‐diffusion model mentioned in Equations (9) and (10), the modified model proposed by Sha et al. is simpler because the PR effect cannot be trivially incorporated in the drift‐diffusion model (attributed to the difficulties of the retrieval of the simulation parameters), although both of them are commonly used theoretical models for investigating device physics of solar cells. They studied the performance of MAPbI_3_ perovskite solar cells with different active layer thickness and demonstrated that different thickness of the active layer played an important role in achieving PR in perovskite solar cells.^[^ ^195^ ^]^

**Figure 16:**
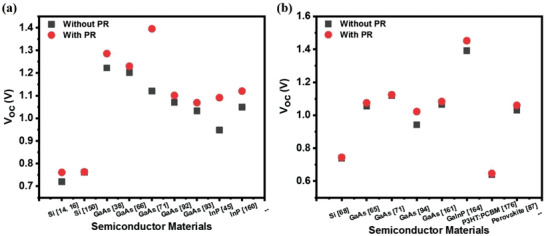
Comparisons of the *V*_oc_ values with and without PR in solar cells using different semiconductor materials from a) theoretical research and b) experimental research. References for the *V*_oc_ values are shown in parenthesis in the *x*‐axis labels.

In conclusion, PR achieved in lasers has the potential to control spontaneous emission and reduce the lasing threshold because of reabsorption. Furthermore, for LEDs, PR plays an important role in assisting with optical coupling and redirecting photons from trapped to outcoupled modes. Based on this effect, the EQE of LEDs can be significantly enhanced due to stronger light outcoupling achieved by the re‐emission of photons generated in the active layer, despite reabsorption typically being regarded as a loss mechanism in LEDs. Finally, the EQE of solar cells can be enhanced through the same mechanism as LEDs and PR is capable of enhancing internal PLQEs of solar cells significantly. In addition, PR is able to reduce the loss of photons both from solar light and from multiple radiative recombination events, so that all photogenerated electron‐hole pairs can be fully separated and transported to different electrodes to improve the performance. **Figure** **16** summarizes the enhancement of *V*_oc_ of different solar cells with and without PR in published theoretical and experimental research. However, the increases of *V*_oc_ from experimental results are not as high as expected compared to the theoretical ones, which is attributed to the idealized situations in the cases of theoretical calculations and simulations and to the relatively fewer experimental studies of PR.

## 6 Summary and Outlook

PR is the process of reabsorption of photons produced from radiative recombination in semiconductors. The result of this process is that new electron–hole pairs can be generated and the carrier population in the semiconductor becomes altered. In many cases PR can improve the optical properties of semiconductor materials and enhance the efficiency of the optoelectronic devices on which they are based. However, there is some ambiguity in the definition of PR that has led to confusion regarding the importance of PR and regarding the impact of PR on device performance parameters. For example, some previous experimental and theoretical studies have attributed increases in both the *V*_oc_ and *J*_sc_ in solar cells to PR. However, this is debatable because the theory suggests only *V*_oc_ is affected by PR, not *J*_sc_. So, while PR can impact the performance of the device, in many cases, other factors are also at play that make it difficult to separate contributions from PR and other light management effects to device performance improvements.

In this review, efforts were made to clearly define PR and to comprehensively present the theory and application of PR in semiconductors. In the past decades, many theoretical and experimental studies have been published that investigate and demonstrate PR in applications of semiconductors to many areas of optoelectronics, especially solar cells. Here, we discussed the basic theory of PR through the principles of radiative recombination and the SQ limit. Owing to the different theoretical expressions and equations associated with PR, it is useful to understand this effect in the context of different applications initially. Additionally, we reviewed different methods that have been used to achieve PR in semiconductor devices. In general, the main approach to achieve PR in the active layer of devices is to increase the pathway for photons from radiative recombination to be reabsorbed. Then, we discussed how PR is characterized in different applications. We described characterization methods such as edge emission spectra, TRPL spectra, output power, LEE, and PCE, which show evidence of PR in thin films, single crystals, and semiconductor devices, including lasers, LEDs, and solar cells (**Table 2** ). As for applications, PR can not only reduce the threshold current of lasers, but can also increase the EQE of LEDs. Furthermore, in the past few decades, solar cells attracted more research interests and PR is more commonly reported for solar cells than for other applications. One of the most important impacts of PR in solar cells is to increase the *V*_oc_ of the devices for both traditional materials (Si, III–V materials) and more novel materials (e.g., perovskite). Thus, we reviewed the expressions and equations that explain how PR affects the *V*_oc_ of solar cells in theory. We also discussed how PR affects the external PLQE to enhance the PCE of solar cells. Compared to Si solar cells and OSCs, PR has been more widely used in III–V solar cells and perovskite solar cells to improve device performance. In particular, fabrication technologies that promote PR in III–V thin‐film solar cells have already been achieved and applied at a large scale. We summarize the different device applications of PR achieved by different methods and their experimental and theoretical improvements in **Table** 4. It covers almost all important and representative work of PR relevant to semiconductor devices. From the current point of view, the methods used in some work to achieve PR in devices may not be optimal. For example, if nanostructured rear‐reflector mirrors were used in some early cases instead of flat rear‐reflector mirrors, the effect of PR may have been more strongly enhanced. However, it is no doubt that these previous results provide a lot of inspiration and experience for future research. It is affirmed that these previous reports have positive and long‐term significance for future research on PR.

**Table 4:** Summary of different device applications of PR and improvements

Type of Devices	Methods	Active Materials	Improvements	Reference No.
Laser	Rear‐reflector mirror	GaAs	Reduce threshold current by 20%	^[^ ^122^ ^]^
	Rear‐reflector mirror	GaAs	Reduce threshold current by 20%	^[^ ^123^ ^]^
	Photonic nanostructure	Ga_0.47_In_0.53_As/InP	Reduce threshold current by up to 40%	^[^ ^80^ ^]^
LED	Rear‐reflector mirror	AlGaAs/GaAs	Increase efficiency from 22% to 27%	^[^ ^50^ ^]^
	Rear‐reflector mirror	InGaN/GaN	Enhance extracted light intensity by more than 65%	^[^ ^57^ ^]^
	Rear‐reflector mirror	GaAs	Increase efficiency from 3% to 12.5%	^[^ ^130^ ^]^
	Photonic nanostructure	Alq3	Increase EQE from 1% to 4%	^[^ ^53^ ^]^
	Nanostructured rear‐reflector mirror	Al_x_Ga_1‐x_As	Increase EQE from 22% to 31%	^[^ ^55^ ^]^
Solar cell	Thick film	Si	Increase efficiency from 27.1% to 29.05%	^[^ ^15^ ^]^
	Thick film	Si	Increase efficiency from 29.05% to 29.43%	^[^ ^16^ ^]^
	Rear‐reflector mirror	Si	Increase efficiency from 25.6% to 26.3%	^[^ ^68^ ^]^
	Rear‐reflector mirror	GaAs	Increase ERE by 150%; enhance *V*_oc_ by 19 mV	^[^ ^65^ ^]^
	Rear‐reflector mirror	GaAs	Increase *V*_oc_ by 28 mV	^[^ ^66^ ^]^
	Rear‐reflector mirror	GaAs	Increase *V*_oc_ by 4 mV experimentally and 275 mV theoretically	^[^ ^71^ ^]^
	Rear‐reflector mirror	GaAs	Increase *V*_oc_ by 18 mV	^[^ ^161^ ^]^
Solar cell		GaAs	Increase *V*_oc_ by 63.5 mV and efficiency by 1.77%	^[^ ^38^ ^]^
	Rear‐reflector mirror	GaInP	Increase *V*_oc_ by 60 mV and efficiency by 1.6%	^[^ ^164^ ^]^
		InP	Increase *V*_oc_ by 143 mV and efficiency by 4.5%	^[^ ^45^ ^]^
	UV‐blocking layer	P3HT:PCBM	Increase *V*_oc_ by 8 mV and efficiency by 0.22%	^[^ ^176^ ^]^
	Rear‐reflector mirror	Perovskite	Increase efficiency from 20.1% to 30.69%	^[^ ^195^ ^]^
	Photonic nanostructure	Perovskite	Increase *V*_oc_ by 30 mV and efficiency from 14.5% to 16.3%	^[^ ^87^ ^]^
	Nanostructured rear‐reflector mirror	GaAs	Increase efficiency from 26.4% to 33.5%	^[^ ^67^ ^]^
	Nanostructured rear‐reflector mirror	GaAs	Increase efficiency from 16.2% to 19.9%	^[^ ^94^ ^]^
	Nanostructured rear‐reflector mirror	Perovskite	Increase efficiency from 20.1% to 30.88%	^[^ ^195^ ^]^

Although achieving PR in semiconductor devices has many advantages, there are still some issues that need to be addressed. Thus, we propose a few research guidelines to achieve more efficient use of PR in future applications. First of all, according to previous research, it is evident that PR is able to play an important role in improving the performance of a wide range of solar cells because it enhances light reabsorption, increases generate rate and increases the *V*_oc_. However, only a few examples show the influence of PR in other applications such as lasers and LEDs. In fact, it is still debatable as to whether PR is helpful for semiconductor devices besides solar cells, and this aspect still needs to be investigated and demonstrated through more applications in light‐emitting devices.

Second, the influence of PR is obvious in III–V solar cells and perovskite solar cells in which the active materials are highly radiative (high luminescence quantum yields). However, there is also potential for PR to be achieved in quantum dot solar cells (QDSCs) and OSCs and even polymer/nanocrystal hybrid solar cells. These latter solar cell types have developed rapidly due to their advantages such as large area coverage, low‐cost fabrication, good film quality, and broad spectral tunability to match the sun's spectrum, but the PCE of these solar cells is still considered too low for commercialization.^[^ ^203^, ^204^, ^205^, ^206^, ^207^, ^208^, ^209^, ^210^ ^]^ In particular, as the radiative efficiency of newer types of solar cells improve, PR will play a more important role in optimizing device efficiency. This is because PR can improve the use of radiatively recombined photons and the difficulty of light trapping in low‐dielectric‐constant photovoltaic thin films can be solved through recent innovations in materials and device fabrication processes. However, the priorities are to clearly investigate and demonstrate the mechanism of PR in QDSC, OSC and other emerging solar cells devices, and to figure out the relationship between PR and the morphologies of the films in these devices. After that, the influence of PR on the optical and electrical behavior of the devices can be further studied and explained thoroughly. As an example, to achieve PR in OSCs, adding a rear‐reflector mirror could be an effective method because rear‐reflector mirrors have already been widely used to study the PR in other solar cell technologies in the past, especially III–V devices. Meanwhile, it is true that rear reflectors are very common in OSCs, but PR achieved by rear reflectors in recent high‐efficiency OSCs (such as fullerene‐free OSC devices) has not been reported yet. Therefore, more work is needed to demonstrate enhancement by PR in organic devices by adding and optimizing rear‐reflector mirrors.^[^ ^211^, ^212^, ^213^, ^214^, ^215^, ^216^, ^217^ ^]^

Third, it is known that incorporating nanostructures in semiconductor devices can facilitate light trapping and light extraction. For example, nanostructured solar cells often exhibit enhanced absorption of sunlight which leads to increased generation of electron‐hole pairs. However, we have discussed that photons can also be internally regenerated by the semiconductor through radiative recombination. Therefore, nanostructures may be helpful for enhancing absorption of the fraction of photons formed through radiative recombination in addition to the direct photons from the sun. For this case, in contrast to enhancing sunlight absorption, the nanostructures should be designed to increase absorption at a narrow wavelength range that corresponds to the energies near the band gap of the semiconductor from which radiative recombination occurs. Therefore, resonant photonic and plasmonic nanostructures may be effective candidates for enhancing PR in optoelectronic devices at specific wavelengths.

Finally, many approaches to achieve PR in this review could be improved to better suit the intended application. Thick active films are the easiest approach to increase reabsorption, but the balance between carrier mobility and the thickness of the film is also a problem in semiconductor devices that limits this approach. Reflectors and nanostructures have been used more in thin‐film semiconductor devices in the recent decades. However, the former one will increase cost because high reflectivity is typically achieved using noble metals or dielectric multilayers. The use of nanostructures for PR includes a large number of candidate structures and materials (e.g., nanoparticles, nanoholes, nanogratings, photonic crystals). Many of these nanostructures have complicated fabrication procedures and requirements for large‐area device applications. Thus, simple and advanced methods need to be found to apply PR in semiconductor devices used in commercial applications.

In conclusion, PR is an effect often used to improve the performance of semiconductor devices. The theory of this effect has been studied over the last several decades and PR can be applied and achieved in a wide range of fields. However, the control and implementation of PR is complex and future improvements using PR will require further study and development in applied settings. The development of more efficient and controllable methods to implement PR will lead to a greater variety of optoelectronic applications for PR in the future.

## Conflict of Interest

The authors declare no conflict of interest.

## Supporting information

Supporting Information
