# Supplementary material for: Photon Recycling in Semiconductor Thin Films and Devices
Source: Adv Sci (Weinh). 2021 Aug 19;8(20):2004076. doi: 10.1002/advs.202004076 (PMC8529496; doi:10.1002/advs.202004076)
Supplement: Supplementary file 1 — Supporting Information [file ADVS-8-2004076-s001.pdf]

© 2021 Wiley-VCH GmbH

ADVANCED SCIENCE
Open Access

Supporting Information

for *Adv. Sci.*, DOI: 10.1002/advs.202004076

Photon Recycling in Semiconductor Thin Films and Devices

Zhongkai Cheng[1], *Deirdre M. O'Carroll**[1,2]

Supplementary Information

Photon Recycling in Organic Semiconductor Films and Devices

Zhongkai Cheng[1] *and Deirdre M. O'Carroll**[1,2]

[1] Department of Chemistry and Chemical Biology, Rutgers University, 123 Bevier Road, Piscataway, New Jersey, 08854

[2] Department of Material Science and Engineering, Rutgers University, 607 Taylor Road, Piscataway, New Jersey, 08854

Table S1: Summary of advantages and disadvantages of different methods to achieve PR in a variety of semiconductor materials and devices.

Method	Advantages	Disadvantages
**Thick Film**	Simple	Low efficiency of devices; Limited area of applications
**Rear-reflector Mirror**	Compact and low-profile; Large area of applications; Long distances for photons for a second or multiple pass at absorption	Limitation of photon absorption
**Photonic Nanostructure**	Diverse nanostructures; Enhancement of light harvesting and trapping	Strong parasitic absorption losses in metals at optical frequencies
**Nanostructured Rear-reflector Mirror**	Diverse nanostructures; Enhancement of light harvesting and trapping; Long distances for photons for a second or multiple pass at absorption	Complex fabrication process
